# High-Throughput Experimentation, Theoretical Modeling, and Human Intuition: Lessons Learned in Metal–Organic-Framework-Supported Catalyst Design

**DOI:** 10.1021/acscentsci.2c01422

**Published:** 2023-01-26

**Authors:** Katherine E. McCullough, Daniel S. King, Saumil P. Chheda, Magali S. Ferrandon, Timothy A. Goetjen, Zoha H. Syed, Trent R. Graham, Nancy M. Washton, Omar K. Farha, Laura Gagliardi, Massimiliano Delferro

**Affiliations:** † Chemical Sciences and Engineering Division, Argonne National Laboratory, Lemont, Illinois60439, United States; ‡ Department of Chemistry, University of Chicago, Chicago, Illinois60637, United States; ¶ Department of Chemical Engineering and Materials Science, University of Minnesota, Minneapolis, Minnesota55455, United States; § Pritzker School of Molecular Engineering, University of Chicago, Chicago, Illinois60637, United States; ∇ Pacific Northwest National Laboratory, Richland, Washington99354, United States; ∥ Department of Chemistry, Northwestern University, Evanston, Illinois60208, United States; ⊥ James Franck Institute, Chicago Center for Theoretical Chemistry, University of Chicago, Chicago, Illinois60637, United States

## Abstract

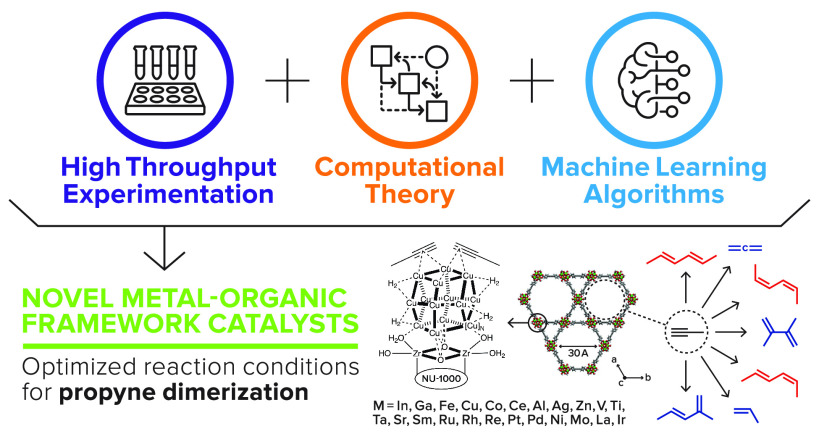

We have screened an array of 23 metals deposited onto the metal–organic framework (MOF) NU-1000 for propyne dimerization to hexadienes. By a first-of-its-kind study utilizing data-driven algorithms and high-throughput experimentation (HTE) in MOF catalysis, yields on Cu-deposited NU-1000 were improved from 0.4 to 24.4%. Characterization of the best-performing catalysts reveal conversion to hexadiene to be due to the formation of large Cu nanoparticles, which is further supported by reaction mechanisms calculated with density functional theory (DFT). Our results demonstrate both the strengths and weaknesses of the HTE approach. As a strength, HTE excels at being able to find interesting and novel catalytic activity; any *a priori* theoretical approach would be hard-pressed to find success, as high-performing catalysts required highly specific operating conditions difficult to model theoretically, and initial simple single-atom models of the active site did not prove representative of the nanoparticle catalysts responsible for conversion to hexadiene. As a weakness, our results show how the HTE approach must be designed and monitored carefully to find success; in our initial campaign, only minor catalytic performances (up to 4.2% yield) were achieved, which were only improved following a complete overhaul of our HTE approach and questioning our initial assumptions.

## Abstract 2

We studied the deposition of 23 different metallic precursors onto the metal−organic framework (MOF) NU-1000 for propyne dimerization to hexadienes in a high-throughput data-driven campaign.

## Introduction

Metal–organic frameworks (MOFs) constitute a class of porous crystalline materials that can act as finely tuned heterogeneous catalyst supports.^1−5^ They can enhance catalysis through confinement or containment effects,^25,6^ support effects, high surface area, and framework modulation of molecular transport.^7,8^ These all represent phenomena that are not present in traditional inorganic metal oxide supports. Through the deposition of catalytically active metal sites onto MOF nodes via atomic layer deposition in MOFs (AIM)^9−12^ or surface organometallic chemistry (SOMC),^13^ the porous MOF NU-1000^14^ has proven to be an effective support for catalytic sites capable of ethylene hydrogenation^10,15^ and oligomerization,^16−18^ propane oxidative dehydrogenation,^19^ alkene epoxidation,^20^ and propyne isomerization.^9^

imprNonetheless, the design of novel MOF catalysts is quite challenging: deposited active sites in MOFs are by their nature quite difficult to characterize,^21^ and the active site and catalytic activity are known to change depending on deposition technique.^19^ As such, conventional computational approaches may struggle to convincingly describe the underlying chemistry, although some success has been demonstrated.^10,18,22^ In particular, MOF-deposited nanoscale catalysts,^23^ especially for highly fluxional metals such as Cu,^11,21,24−27^ provide a very difficult challenge for theory despite their versatile reactivity. On top of this, the activity of MOF catalysts can vary heavily due to synthetic or operating parameters, such as metal weight loading, reduction temperature, reaction temperature, space velocity, and operating pressures—all difficult parameters to model via standard theoretical means (i.e., density functional theory (DFT)). Thus, high-throughput experimentation (HTE) appears well-positioned to play an important role in MOF catalyst design, as it can rapidly explore chemical space with minimal (i.e., data-driven or machine-learned) theoretical input. Despite this, high-throughput approaches are quite rare in experimental studies of metal–organic frameworks^28−31^ and in catalytic applications of MOFs.^9^

**Figure 1:**
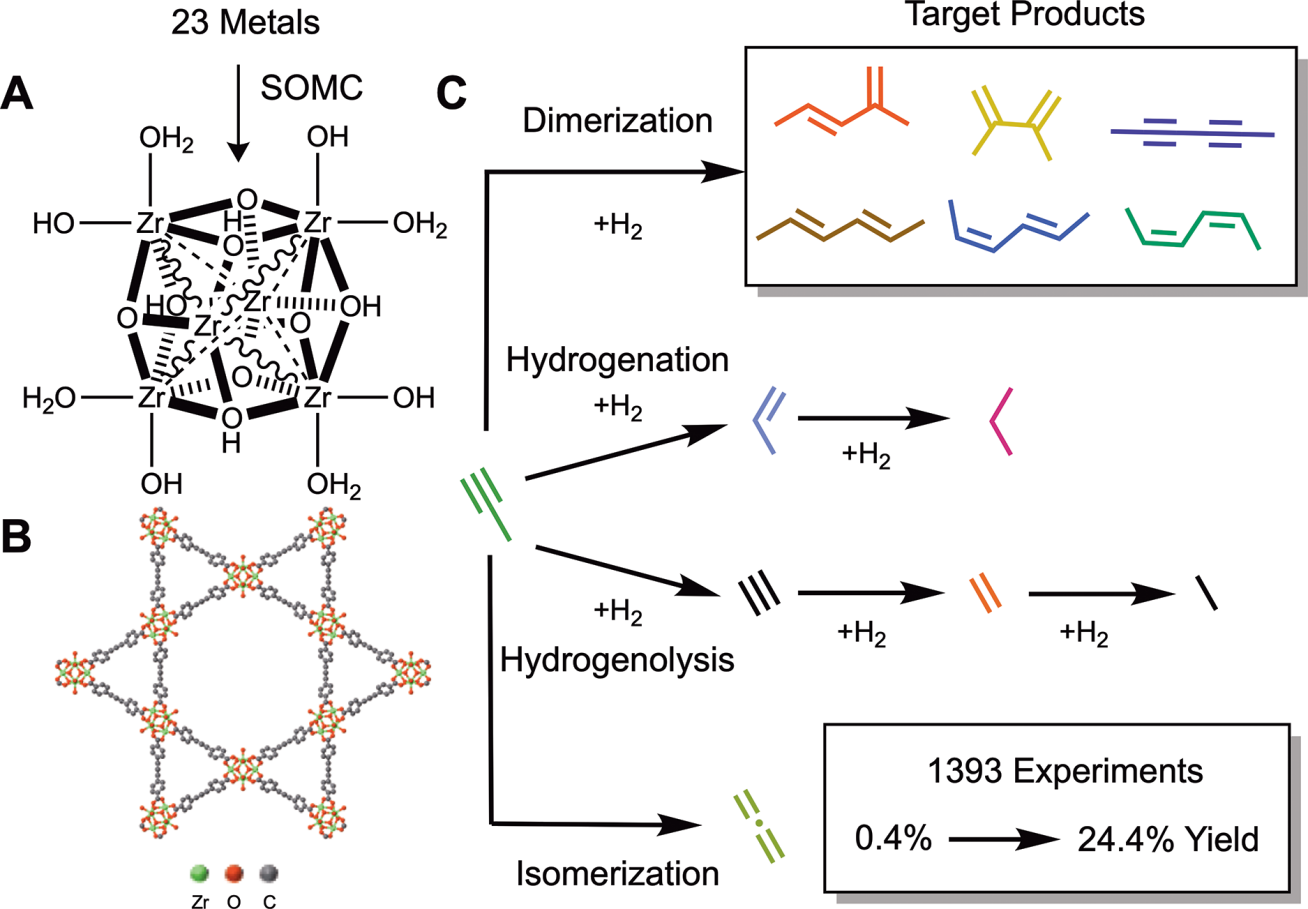
(A) Chemical schematic of the Zr_6_ (μ_3_-OH)_4_ (OH)_4_ (OH_2_)_4_ NU-1000 node, with oscillating and dashed bonds showing the locations of the secondary binding unit linkers. (B) Crystallographic visualization of NU-1000.^32^ (C) Product pathways for propyne dimerization, hydrogenation, hydrogenolysis, and isomerization.

In this work, we present the first application of this high-throughput approach to developing MOF-supported catalysts. By empirically learning the interaction between synthetic and operating conditions over 23 different metals, we use data-driven algorithms to identify and optimize interesting catalytic activity prior to in-depth characterization and theoretical modeling. Focusing on the reaction of propyne dimerization, important for the removal of alkyne compounds from polymerization feedstocks,^33,34^ we are able to improve hexadiene yields in flow reactors from 0.4 to 24.4% on the MOF NU-1000 (Figure 1). Characterization of the final optimized catalysts reveals the necessity of Cu nanoparticle formation in the MOF for achieving significant catalytic activity, which is supported by theoretical results.

However, these results were not obtained without significant intellectual effort—initial assumptions regarding the nature of metal active sites and the range of relevant experimental variables proved incorrect, resulting in over half the experiments conducted giving no significant hexadiene yield. Improvement was only obtained through questioning our initial assumptions and a redesign of our high-throughput campaign, with the full story only revealing itself through careful posthoc characterization and modeling of the successful catalyst. Thus, our results show how HTE is not a fully automated approach—although useful for finding unexpected catalytic activity, high-throughput campaigns often must be monitored and redesigned to find success.

## Results and Discussion

Our initial search space consisted of 22 different metals, due to their known propensity to catalyze alkyne functionalization and upgrading (Ag, Al, Ce, Cu, Fe, Ga, In, Ir, La, Mo, Ni, Pd, Pt, Re, Rh, Ru, Sm, Sr, Ta, Ti, V, and Zn), deposited onto NU-1000 at five different weight loadings (0.5–5 wt %) and tested in a 16-reactor parallel plug flow reactor (Flowrence from Avantium). In addition to the metal identity and loading, five different reaction variables were also identified. These included two pretreatment variables related to the reducibility of the organometallic complexes to the active metal site, hydrogen reduction temperature (150–225 °C) and steamed air treatment temperature (125–225 °C) used to remove residual organic material, and operating conditions that influence conversion and selectivity including total flow (2.5–20 mL/min), which in turn will affect the space velocity, reaction temperature (100–250 °C), and the amount of H_2_ cofed during the reaction (0– 5 vol %). These initial parameters were chosen based on domain knowledge^9,16,35−38^ and in consideration of the thermal and chemical stability of NU-1000.^15,39^

Given this search space, our initial selection algorithm employed Bayesian optimization^40−45^ to maximize the total hexadiene yield (defined as the ratio of moles of hexadiene formed to the number of moles of propyne that have been consumed) for each metal individually, prioritizing In and Cu over other metals due to literature precedent.^9^ A summary of this approach is given in Figure S1. However, after several iterations of this approach totaling 721 experiments (taking roughly 6 months of work), the results showed very little activity or selectivity for any metal in the parameter space outlined, obtaining a maximum hexadiene yield of 4.2% over 4 wt % Cu/NU-1000 (H_2_ reduction *T* = 150 °C, *T*_rxn_ = 250 °C, space velocity = 2000 mL/min/g_cat_, 5 vol % H_2_ cofed) and little improvement of predictions with number of iterations. Furthermore, Cu was far-and-away the best-performing catalyst, with the next-highest yields obtained with Ga (1.0%) and In (0.4%). Thus, a decision was made to abandon the initial approach.

Prior studies have shown that an increase in H_2_ partial pressure in the inlet results in a higher selectivity to propylene, and that oligomers are preferentially produced at lower H_2_:C_3_H_4_ ratios in rather large concentrations,^35−37,46^ and yet, this trend was not observed in our initial results. Instead, the highest hexadiene yields were obtained at the maximum 5 vol % H_2_ concentrations. Based on this unexpected trend, we made the decision to screen all metals (slightly biased toward Cu, based on its larger activities) at a higher concentration of H_2_ (40 vol %, SI Section 1.2). To our surprise, this immediately boosted hexadiene yields on Cu from 4.2 to 15.4%. This key result allowed us to redesign our high-throughput campaign to focus on Cu activity and higher inlet H_2_ concentrations (0–80%) while eliminating pretreatment variables (air temperature and H_2_ reduction temperature), which did not seem to have an impact on hexadiene yields.

**Figure 2:**
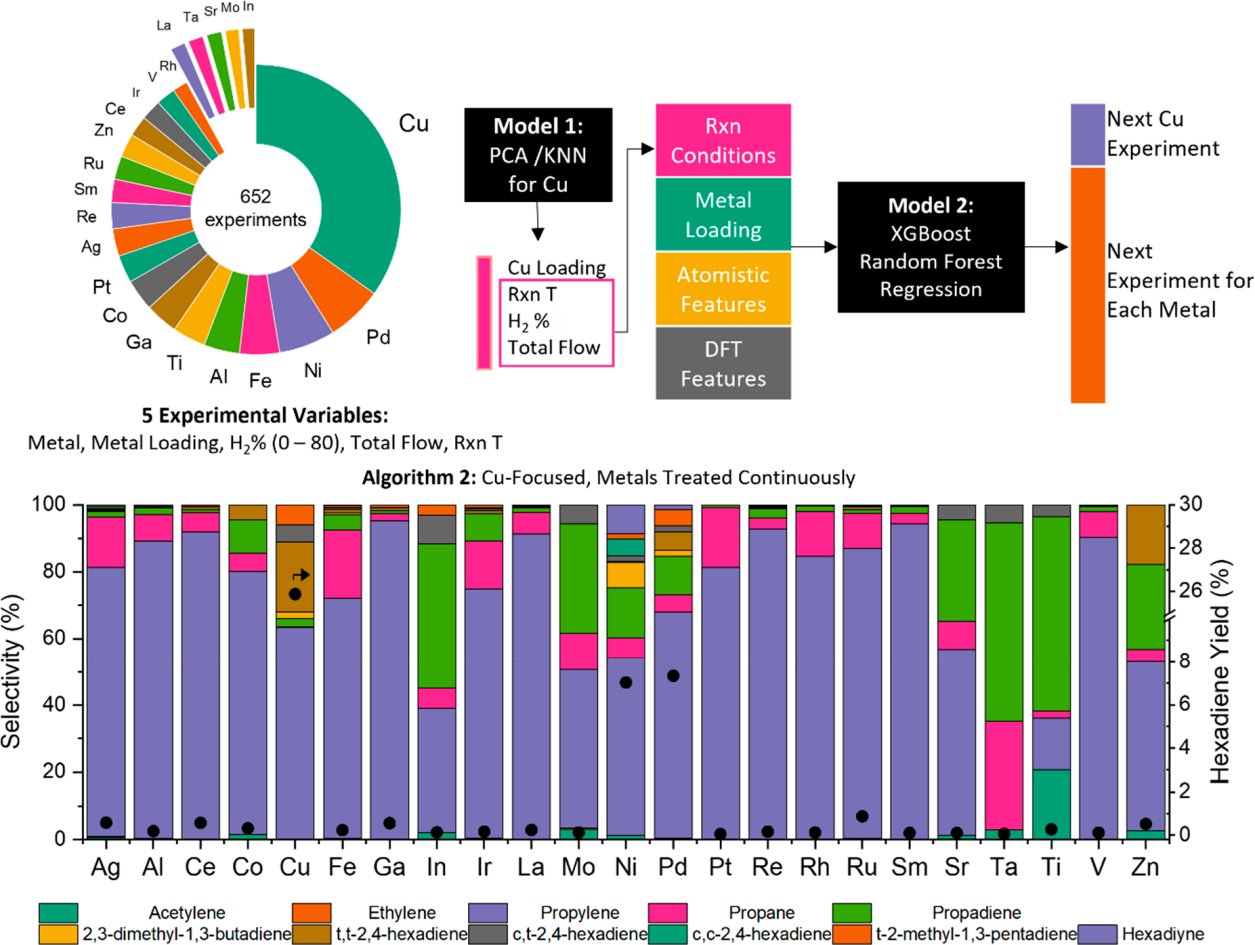
Summary of the second campaign to optimize hexadiene yield over 23 different catalytically active metals. The distribution of metals experimentally tested is shown in the pie chart (expanded for clarity) and totaled 652 experiments. The dual-model scheme is outlined utilizing principal component analysis (PCA) and k-nearest neighbors (KNN) to first optimize Cu catalyst conditions, after which XGBoost random forest regression is used to make predictions for each metal. The bottom graph indicates the highest hexadiene yield (black circles) for each metal and the associated product selectivities.

Figure 2 summarizes the approach used in our redesigned campaign. Instead of optimizing conditions for all metals symmetrically, a dual-model scheme was employed, in which model 1 selects operating parameters (loading, reaction temperature, inlet H_2_ concentration, and space velocity) for Cu and model 2 selects other metals and metal loadings to test at these operating conditions. This approach enabled both (i) a more efficient use of the high-throughput reactor bed and (ii) the possibility of learning trends across different metals with model 2. To achieve this latter goal, the adsorption of propyne and H_2_ onto different single-atom catalysts deposited onto NU-1000 was computed with DFT. Thermochemical features of these adsorptions (e.g., Δ*E*_*ads*_, Δ*H*_*ads*_, Δ*G*_*ads*_) were combined with atomic data^47,48^ of different metals to interpolate between different metals. Full details of these models are available in the Supporting Information.

Using this approach, yields on Cu were increased from the 4.2% found in our initial campaign to 24.4% over the course of 227 experimental trials at 79 unique operating conditions (with several conditions tested multiple times to ensure reproducibility). Furthermore, significant yields of 7.3 and 7.0% were obtained for Pd and Ni, respectively, showing the ability of our second approach to discover interesting reactivity on new metals while simultaneously optimizing Cu. Interestingly, the highest hexadiene yields for Pd and Ni were found at considerably different conditions. Pd had the highest yield at 5 wt %, 200 °C, 460 mL/min/g_cat_, and 13.5 vol % H_2_ cofed, while Ni had the highest yield at 3 wt %, 175 °C, 162 mL/min/g_cat_, and 38.5 vol % H_2_ cofed. These in turn also differ dramatically from Cu, which exhibited high yields with much higher space velocities, between 1000–2000 mL/min/g_cat_, and lower metal loading.

**Figure 3:**
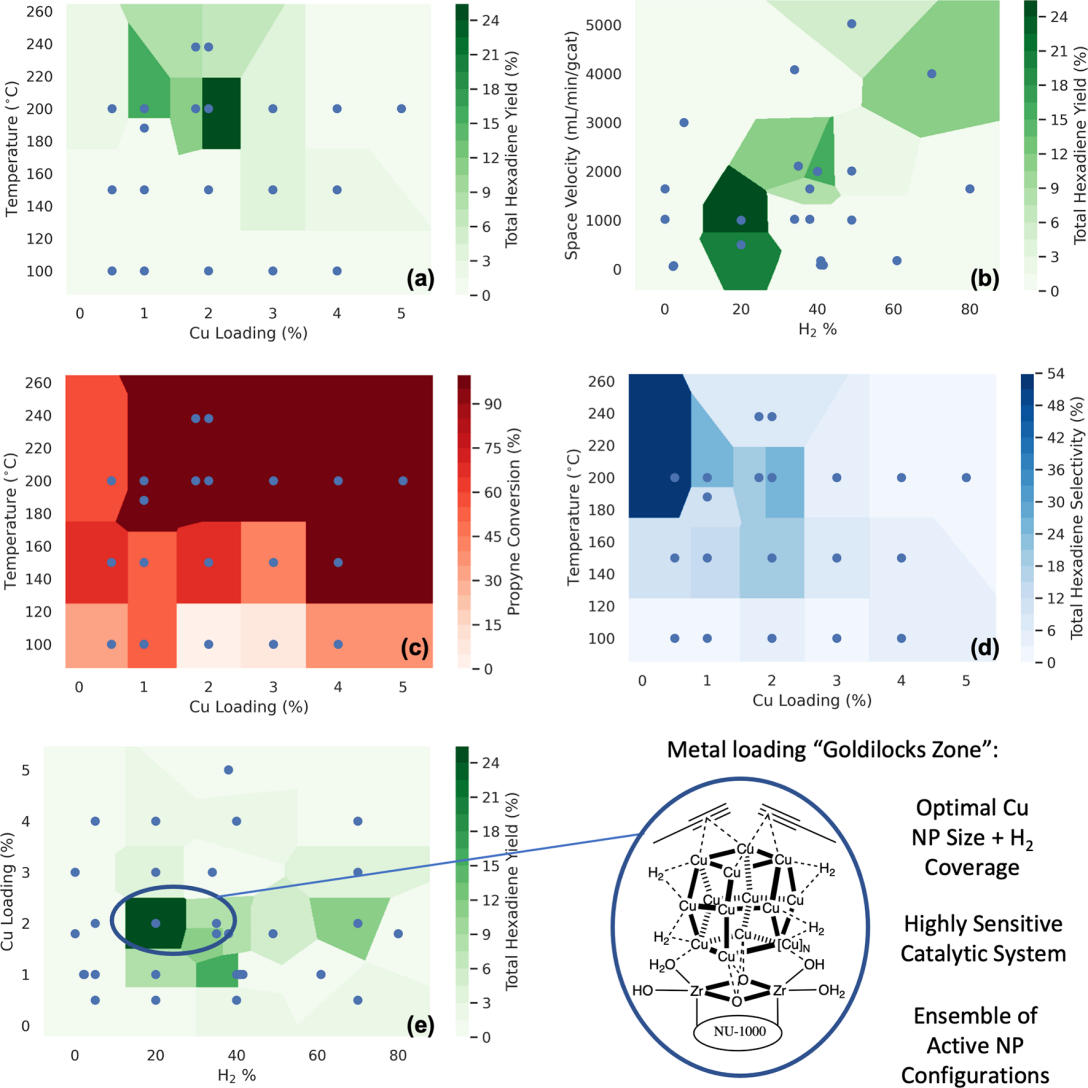
Different Voroni tesselations (KNN models with *K* = 1) of the maximum hexadiene yield, conversion, and selectivity achieved in two-dimensional cuts of experimental parameter space (metal loading, H_2_ vol %, space velocity, and reaction temperature) for the 79 trial-averaged Cu experiments carried out in the second high-throughput campaign, represented by blue marks on each plot. (a) Cu loading vs temperature (yield), (b) space velocity vs H_2_ vol % (yield), (c) Cu loading vs temperature (conversion), (d) Cu loading vs temperature (selectivity), and (e) H_2_ vol % vs loading (yield). Each set of plots demonstrates a characteristic of the final data set. The nanoparticle structure shown next to Figure 3e is meant only as a schematic and not as an existing species in the active catalyst; actual nanoparticle sizes are shown in Figure 4.

Figure 3 shows different Voroni tesselations (K-nearest-neighbor models with *K* = 1) of the maximum hexadiene yield (green), conversion (red), and selectivity (blue) achieved in two-dimensional cuts of experimental parameter space (metal loading, H_2_ vol %, space velocity, and reaction temperature) for the 79 trial-averaged Cu experiments carried out in our second campaign. Each set of plots demonstrates one trend found in the final data set. The first set of plots, showing yield as a function of loading and temperature (Figure 3a) and H_2_ vol % and space velocity (Figure 3b), emphasizes the sparsity of the catalytic activity observed in the experimental parameter space. High catalytic activity for Cu requires a highly specific reaction temperature and loading as well as space velocity and H_2_ vol %, and small changes in any of these parameters can drastically affect results. Interestingly, a trend in catalytic activity appears when H_2_ vol % and space velocity are increased in turn with each other (i.e., increasing one or the other alone results in diminished yields).

Figure 3c,d demonstrates the trade-off observed between conversion and selectivity as loading and temperature are changed. Higher conversions are noted at the intersection of high temperature and high metal loading. However, higher selectivity for hexadienes is observed at higher dispersion of metals. Thus, the optimal yields occur at the combination of high temperatures (200 °C) and medium metal loadings (2 wt %). This demonstrates the importance of considering conversion and selectivity in tandem for the optimization of yield, as the conditions that promote formation of one product may not be thermodynamically favorable. Interestingly, this trade-off appears to occur differently for Ni and Pd, which is discussed in the Supporting Information (Figure S7).

Finally, Figure 3e shows the highly dependent behavior of the catalytic system (M + MOF) on both loading and H_2_ vol %. These dependencies point strongly toward nanoparticle-like catalytic behavior, in which one needs a large enough nanoparticle so that two propyne molecules can react on its surface but small enough such that excess H_2_ is not adsorbed to over hydrogenate the reactants. If one has in mind separated metal centers in the MOF, which carry out catalysis independently from one another, then yield should increase roughly linearly with loading, up to a point at which the increased loading results in sintering or other destructive processes. Instead, a sharply peaked activity at a loading of 2 wt % was observed. This implies the existence of a strongly optimal loading, which is difficult to explain without including interactions between Cu sites in one model, most likely through nanoparticle formation.

Indeed, postreaction microscopy analysis of the Cu/NU-1000 catalysts shows the formation of nanoparticles, which is unique to the Cu/NU-1000 catalysts and not exhibited for the other deposited metals. SOMC produces highly dispersed metal species on supports, and XRD analysis of Cu/NU-1000 prior to reaction or preactivation indicates that the Cu species are below the limit of detection for XRD for a catalyst containing 1.52 wt % Cu by inductively coupled plasma-optical emission spectroscopy (ICP-OES, nominal loading of 1.0 wt %). Migration of isolated Cu sites has been previously reported on NU-1000^8,11,21^ where the size of the nanoparticle formed is constrained by the size of the hexagonal (3 nm) and triangular (1 nm) channels of the pore when installed through AIM. Here, the Cu particle size appears to be manipulated by controlling the duration of the time, during which the catalysts are reduced under H_2_.

**Figure 4:**
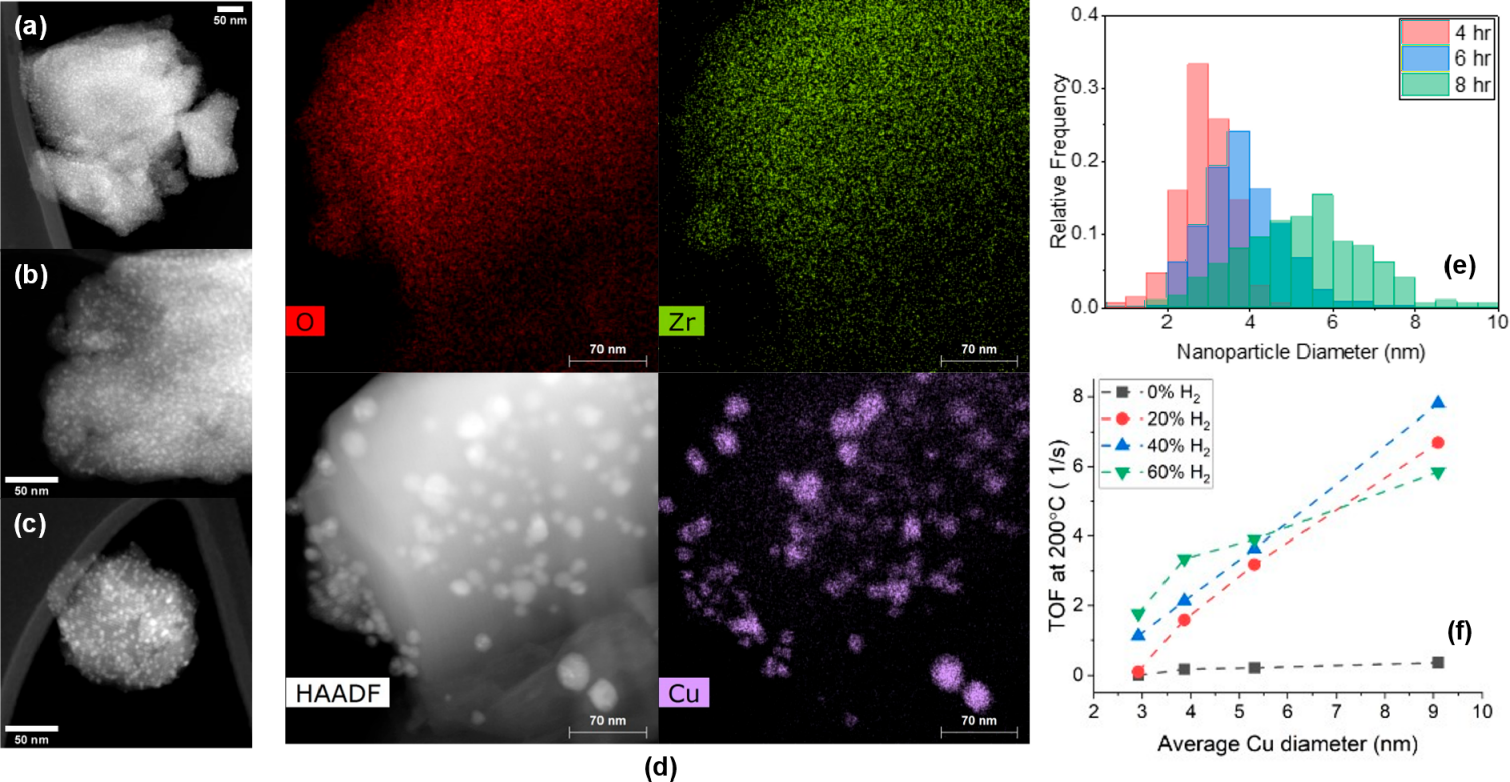
HAADF-STEM of Cu/NU-1000 after (a) 4 h reduction, (b) 6 h reduction, and (c) 8 h reduction in 100 vol % H_2_ at 200 °C and (d) after reaction. (e) Particle size distribution for Cu/NU-1000 after reduction in H_2_ for 4 h (*N* = 376, σ = 0.62), 6 h (*N* = 340, σ = 0.95), and 8 h (*N* = 323, σ = 1.49). (f) Turnover frequency (TOF) at 200 °C of Cu/NU-1000 as a function of Cu nanoparticle diameter calculated from STEM imaging. Reaction conditions: 2% propyne/Ar in 0–60 vol % H_2_, *T* = 100–200 °C, SV = 2000 mL/min/g_cat_ and *P* = 1 atm.

Figure 4 shows the size and distribution of the Cu-supported nanoparticles after (a) 4 h reduction, (b) 6 h reduction, and (c) 8 h reduction, and (d) EDX mapping after a reaction lasting 15 h. The average nanoparticle size grows from subnanometer after 2 h reduction, to 2.92 nm after 4 h, to 3.86 nm after 6 h, and to 5.31 nm after 8 h of exposure to H_2_ (Figure 4e). During the reaction, the particle size continues to increase due to cofed H_2_, increasing to an average of 13.9 nm (Figure 4d). This nanoparticle size greatly exceeds the pore size of the MOF, implying the formation of Cu nanostructures spanning several MOF pores. Both the effect of particle size and inlet H_2_ concentration on turnover frequency (TOF) at 200 °C were explored (Figure 4f). In all cases, larger Cu nanoparticles and higher H_2_ vol % cofeed resulted in a more active catalyst, up to 40%, at which point the TOF was observed to decrease slightly. No other higher oligomers were observed as evidenced by the H balance close to 100% (Figure S11). Yield for hexadiene is greater than that of Cu nanoparticles deposited onto SiO_2_ via SOMC (Figure S13), despite the presence of coke on the Cu/NU-1000 (Figure S15). This higher yield appears to be the result of NU-1000 stabilizing larger nanoparticles—a high-angle annular dark-field scanning transmission electron microscopy (HAADF-STEM) image of Cu/SiO_2_ taken after the reaction shows smaller particle size (Figure S14). Further studies are being conducted to determine the growth kinetics of these nanoparticles and whether nanoparticles may exist within the pores of the MOF or decorate the exterior.

**Figure 5:**
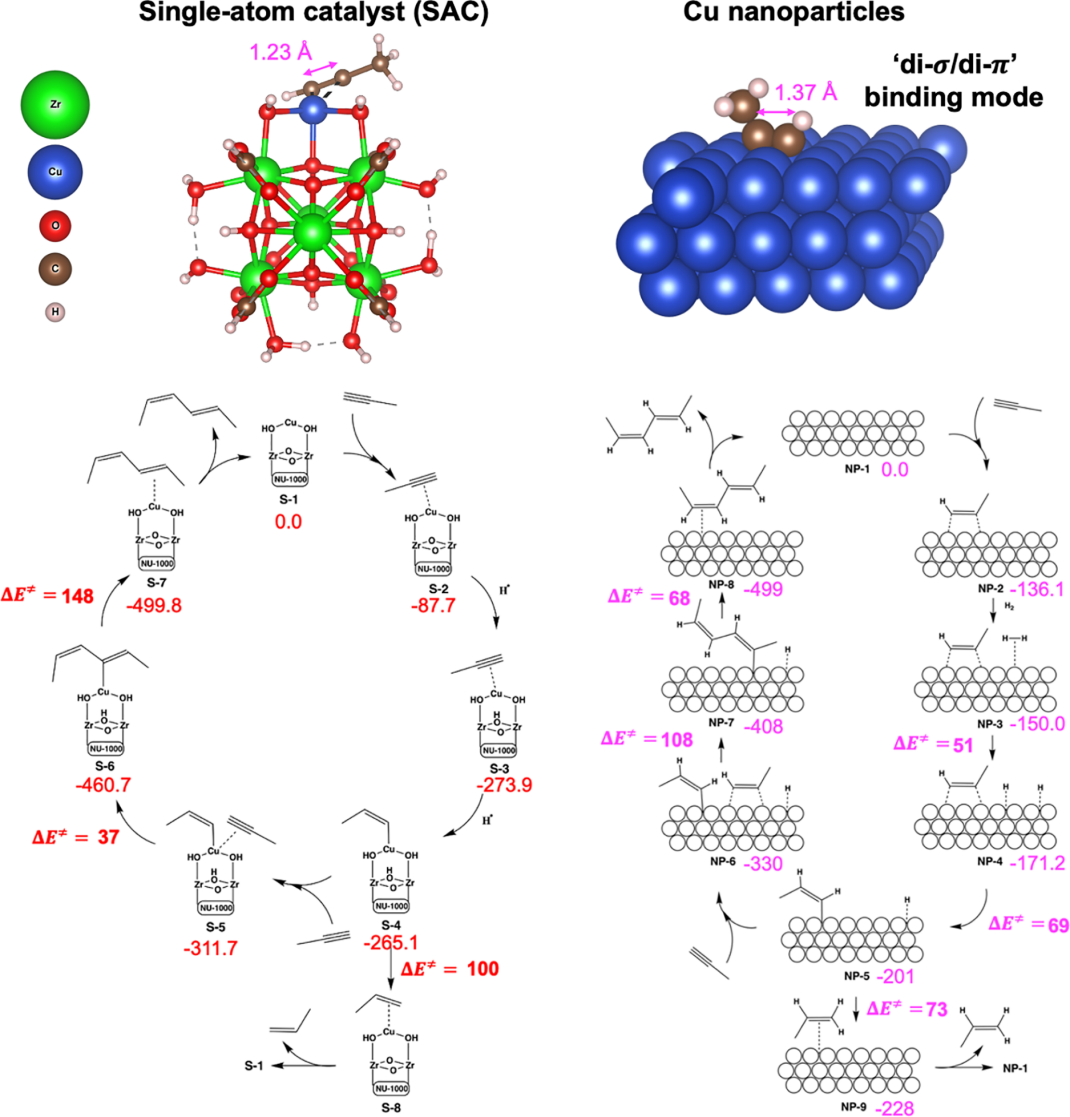
*Top*: Comparison of calculated propyne adsorption conformations on a single-atom catalyst (SAC) model of Cu/NU-1000 to a nanoparticle (NP) model (estimated as a Cu slab). Bottom: Reaction mechanism for propyne dimerization to hexadienes and hydrogenation to propene on the single-atom Cu/NU-1000 (left) and Cu nanoparticles (right). The computed relative electronic energies in kJ mol^–1^ along the reaction mechanism on the two models are shown in red and magenta, respectively.

Finally, to rationalize the high performance of Cu nanoparticles in comparison to single-atom (low% loading) Cu catalysts, reaction mechanisms for propyne dimerization and hydrogenation were computed using DFT (Figure 5). Specifically, dimerization vs hydrogenation reaction pathways on a single-atom catalyst (SAC) model of Cu/NU-1000 (Figure 5, left) were compared to a nanoparticle (NP) model (Figure 5, right, estimated as a slab). These two models are meant to represent the complementary extrema of the active site behavior of polydisperse Cu nanostructures present in our active catalyst (Figure 4d). These two models are not meant to be representative of the polydisperse Cu nanostructures present in our active catalyst but rather of complementary extrema of active site behavior; both models have been utilized previously to model Cu single-atom^49^ and NP catalysts^50^ in the literature.

Following the SAC model first, propyne can adsorb onto the Cu SAC and partially hydrogenate to form intermediate **S-4**; this intermediate can either undergo a second hydrogenation to form propene (**S-8**, Δ*E*‡ = 100 kJ mol^–1^) or undergo C–C insertion with another adsorbed propyne to form hexadiene (**S-7**, Δ*E*‡ = 148 kJ mol^–1^). Likewise, the nanoparticle model intermediate **NP-5** can either undergo further hydrogenation to form propene (**NP-7**, Δ*E*‡ = 73 kJ mol^–1^) or undergo C–C bond formation to form hexadiene (**NP-8**, Δ*E*‡ = 108 kJ mol^–1^). Thus, although hydrogenation is favored in both catalysts, consistent with the observed activity (Figures S1 and S2), hydrogenation is favored by 48 kJ mol^–1^ on the SAC model and by only 35 kJ mol^–1^ on the NP model. This suggests that the mechanistic explanation for the higher hexadiene yield observed for larger Cu loadings and H_2_ cofeed% is that higher H_2_ cofeed assists in formation of NPs in NU-1000, which in turn have increased selectivity toward hexadienes compared to SAC Cu/NU-1000, thereby increasing the overall hexadiene yield. Beyond this, coverage and confinement effects^8^ of the MOF may also play important roles in engendering hexadiene selectivity.

## Conclusions

In this work, we have screened an array of 23 metals deposited onto NU-1000 for the catalytic dimerization of propyne to hexadienes under multiple reaction conditions. Using data-driven algorithms and high-throughput experimentation, hexadiene yields on Cu/NU-1000 were improved in series from 0.4 to 15.4 to 24.4%. Characterization of the catalyst reveals that selectivity toward hexadiene is likely due to large Cu nanoparticles, embedded in the MOF framework, which grow in size when exposed to H_2_ under reductive conditions. This activity is supported by DFT calculations comparing single-atom models of the Cu active site to nanoparticle models, which predict nanoparticles to be more selective for dimerization to hexadienes.

To the best of our knowledge, this project serves as the first application of the HTE approach to post-modification catalysis in MOFs and provides key takeaways for further research in this direction. It is clear that the improved activity obtained in this synergistic study could not have been discovered *a priori*; the high-throughput approach was critical to finding the precise experimental and synthetic conditions at which the catalysts showed meaningful selectivity and conversion to hexadiene. The discovered trend of large amounts of cofed H_2_ drastically increasing hexadiene yields goes against both literature precedent^35−37,46^ and chemical intuition, which dictates that lower H_2_:C_3_H_4_ ratios should facilitate hexadiene formation. This trend could only be explained via post-hoc analysis with microscopy and DFT, which shows that high H_2_% cofeed encourages nanoparticle formation, which is more selective for propyne dimerization than single-atom copper sites. Furthermore, our initial single-atom models of the active site proved at odds with the final nanoparticle catalysts, and even if our DFT models had been more in line with this final characterization, it is unlikely that any *ab initio* model could satisfyingly incorporate the effects of reaction temperature, metal loading, H_2_ cofeed%, and space velocity, all of which proved vital for optimizing hexadiene yield. It is worth noting that features of these single-atom models were utilized in the machine learning scheme developed to select new experiments (Figure 2), which likely hindered their contribution to improving catalyst yield.

On the other hand, this does not mean the HTE approach is void of complications. Designing and initiating a HTE campaign requires committing to a parameter space ahead of time which may or may not contain significant results. Although one attempts to design their campaign wisely to maximize the chances of success, using literature precedent and chemical intuition to select the choices and ranges of the experimental variables, initial assumptions can be proven wrong. For example, literature precedent dictated that a small H_2_ vol % would encourage hexadiene selectivity, which led us to select an initial parameter range of 0–5 vol % H_2_. This unfortunate initial choice led to about six months of work (over half the experiments conducted in this project) resulting in very low hexadiene yields. It required us questioning our initial assumptions and following the data to drastically expand the range of hydrogen concentrations being tested in order to find significant catalytic activity. This overhaul of our HTE approach also provided an opportunity to drop insignificant variables and redesign our experimental selection algorithm, which greatly increased the efficiency of our second campaign. Furthermore, HTE alone cannot be used to explain the catalytic activity it finds—the data must be analyzed, characterization must be done, and the active site must be modeled to provide a full and satisfying picture. We suggest that high-throughput campaigns in general may benefit from a series of low-commitment exploratory searches followed by increasingly targeted and refined searches once interesting behavior has been found.

Thus, our story shows that although data-driven HTE can successfully be used as a tool to discover novel and high-performing materials, it is by no means a fully automated approach. Much care and thought must be put into the design and operation of the campaign throughout its lifetime, and the campaign must be monitored and redesigned if it is not providing satisfactory results. Of course, the parameter space of any HTE campaign can in principle be expanded *ad infinitum* to include all of chemical space (at exponentially increasing cost), but in reality, some (potentially ill-founded) assumptions must be made, and chemical intuition must be applied to design an initial search space and monitor its success. Human intuition and input will continue to be vital to the success of improving catalytic yields. Finally, we hope that this journey shows that it is important to share both successful and unsuccessful results to advance knowledge as a community.

## Methodology

### Metal Deposition

NU-1000 was synthesized as previously described^9,32^ and used as prepared. An automated synthesis platform (CM3 Core Module deck, Unchained Laboratories Inc.) housed in a custom-built N_2_-filled glovebox (MB 200B, MBruan) was used for catalyst synthesis. The CM3 performed both solid and liquid dispensing within a 0.5% tolerance. A surface organometallic chemistry (SOMC) process was used for grafting metals onto NU-1000.^13^ This synthetic approach was used over ALD^9^ and other cation exchange techniques^49^ due to its generalization to many different precursors and feasibility in a high-throughput environment. In detail, 20 mg of NU-1000 was first dispensed into 4 mL vials. Various organometallic precursors (trimethylaluminum, gallium(II) acetylacetonate, trimethylindium, titantium tetraisopropoxide, tetrakis(ethylmethylamino) vanadium(IV), bis(ethylcyclopentadienyl) manganese, bis(*N*,*N*′-di-*t*-butylacetamidinato) nickel(II), bis(dimethylamino-2-propoxy) copper(II), diethylzinc, bis(*t*-butylimido) bis(dimethylamino) molybdenum(VI), methylcyclopentadienyl)(1,5-cylooctadiene) iridium(I), (trimethyl)methylcyclopentadienyl platinum(IV), bis(ethylcyclopentadienyl) ruthenium(II), rhodium(II) acetylacetonate, palladium(II) hexafluoroacetylacetonate, trimethylphosphine(hexafluoroacetylacetonato) silver(I), tris(2,2,6,6-tetramethyl-3,5-heptanedionato) lanthanum(III), tris(2,2,6,6-tetramethyl-3,5-heptanedionato) samarium(III), pentakis(dimethylamino) tantalum(V), or methyltrioxorhenium(VII)) were then dispensed into each vial correlating to either 0.5, 1.0, 2.0, 4.0, or 5.0 wt % metal, after which 4 mL of dried toluene was added. The vials were then placed manually onto a heated unit atop a shaker plate and left to metalate for 72 h at 60 °C and 400 rpm. After cooling, the samples were washed in toluene and centrifuged (Speedvac Concentrator, SPD121P, ThermoElectron) five times. Solvent exchange using pentane was done in the last washing step. After removal of the supernatant fluid, the vials were then removed from the glovebox and vacuum-dried at 60 °C overnight.

### High-Throughput Screening

Dimerization reactions were performed in a 16-reactor fixed bed flow system (Flowrence, Avantium). Typically, 5 mg of as-prepared catalyst was loaded into a quartz reactor (inner diameter = 2 mm, outer diameter = 3 mm, length = 300 mm). Reactions were performed between 100 and 250 °C at a heating rate of 10 °C/min at 1 atm. All gases were purchased from Airgas. Propyne (2 vol %) in Ar was used with pure H_2_ in various ratios for hydrogenation reactions. All catalysts were reduced in 100 vol % H_2_ for 2 h at 200 °C. For each data point collected, the catalysts were first exposed to the propyne/H_2_/Ar mixture for 1 h before measurements, after which two or three measurements were taken per condition tested, and reported values are the average of these data points. N_2_ (UHP) was added to sweep gas to the gas chromatograph (GC) after reaction. He (UHP) was used as an internal standard. The effluent of each reactor was collected in a unique vial for each reactor to avoid cross contamination of effluent streams. The effluent was then analyzed with GC (7890B, Agilent Technologies), equipped with a thermal conductivity detector (TCD) and two flame ionization detectors (FIDs). The gas products identified were methane, ethane, ethylene, propane, propylene, propadiene, acetylene, 2,3-dimethyl-1,3-butadiene, t,t-2,4-hexadiene, c,c-2,4-hexadiene, c,t-2,4-hexadiene, t-2-methyl-1,3-pentadiene, and 2,4-hexadiyne.

### Microscopy

Nanoparticle imaging was conducted using a FEI Talos F200X high-angle annular dark-field scanning transmission electron microscope scanning transmission electron microscope (HAADF-STEM) operated at 200 kV. This microscope was used in coordination with the Center for Nanoscale Materials (CNM) at Argonne National Laboratory. In preparation for analysis by electron microscopy, powdered samples (∼20 mg) were sonicated in ethanol (10 mL) for 15 min. The resulting suspension was dropcast onto a lacey carbon TEM grid (Ted Pella, Inc., UC-A on Lacey 400 mesh Cu). Particle size was measured using ImageJ software.

### Single-Atom Catalyst Model

A cluster model representing a single Zr_6_O_8_ (OH)_4_ (H_2_O)_4_ node of NU-1000 was extracted from a previously optimized unit cell of NU-1000. The hydroxy and aqua ligands on one face of the node were used to deposit different catalytic transition metals, varying in their oxidation state from +1 to +4. The transition metals were ligated to the appropriate number of hydroxy ligands to maintain the overall neutrality of the MOF.^51^ The linkers connecting this node of NU-1000 to the adjacent nodes were truncated as formate groups. The carbon atoms of the formate groups were spatially frozen to mimic the structural rigidity of NU-1000. These cluster models representing single-atom catalytic metals supported on NU-1000 (M-NU-1000) were then employed to compute the adsorption energies of the reactants (propyne, H_2_) on the catalytic metal centers and to study the reaction mechanism for propyne dimerization occurring on a single-atom Cu-NU-1000.

The above cluster models were optimized at the density functional theory level in the *Gaussian 16* software^52^ program using the M06-L^53^ local exchange-correlation density functional. The def2SVP basis set was used for O, C, and H, while the def2TZVPP basis set was used for the catalytic metals and the support metal (Zr).^54^ The SDD effective-core pseudopotential was used for metals present beyond the fourth row of the periodic table to enhance the computational efficiency. Vibrational frequencies were computed in the rigid rotor harmonic oscillator (RRHO) approximation for the optimized structures to determine the nature of the stationary point and to compute the entropic contribution to the free energies. No imaginary frequencies were determined for the intermediates (minimum on the potential energy surface), while only one imaginary frequency along the reaction coordinate was determined for the transition states. All frequencies below 50 cm^–1^ were corrected to 50 cm^–1^ to avoid errors due to anharmonicity.^55,56^ Different possible spin states corresponding to the oxidation state of each transition metal were considered, and only the spin states lowest in energy are reported here.

### Nanoparticle Model

Periodic density functional theory calculations to study the reaction mechanism of propyne dimerization on Cu NP model were performed in the *Vienna Ab Initio Simulation* software (VASP 5.4.4).^57,58^ The Cu nanoparticles formed in NU-1000 upon pretreatment in H_2_ were modeled using a 5 × 5 × 3 Cu(111) periodic slab (75 Cu atoms) with a lattice constant of *a* = 2.522 Å. Such periodic models have previously been used to model large Cu nanoparticles (NPs) in NU-1000.^50^ An additional vacuum of at least 18 Å was used along the *z*-direction. The Perdew–Becke–Ernzerhof (PBE)^59^ exchange-correlation density functional was used along with Grimme’s D3 correction with zero damping for dispersion energy correction.^60^ A plane-wave basis set with a cutoff energy of 400 eV was employed. All structures were optimized using an energy and force convergence criteria of 10^–4^ eV and 0.08 eV/Å respectively. A 3 × 3 × 3 Γ-centered *k*-points grid was used for the Brillouin zone sampling. The climbing image nudged elastic band (CI-NEB) method^61^ with at least eight images between the optimized reactant and product along the reaction coordinate was used for locating the transition state. All calculations were performed without any spin polarization. The electronic energies of the optimized structures were used to represent the potential energy interface along the reaction cycle. The relative energies were calculated using eq 1. The energies of the reference gas phase molecules were obtained by placing a single gas phase molecule at the center of a simulation box having the same dimensions as the Cu NP model. The absolute and relative electronic energies are reported in Table S5.
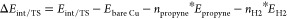
1

## Supporting Information Available

Full description of machine learning models used to accelerate the maximization of hexadiene yields. Optimized coordinates of key intermediates and transition states in the SAC, nanoparticle, and machine learning models. Further experimental characterization of the Cu/NU-1000 catalyst (PDF)
XYZ coordinate files for computational assessment to support mechanistic discussion (ZIP)

## References

[ref1] Lee J.; Farha O. K.; Roberts J.; Scheidt K. A.; Nguyen S. T.; Hupp J. T. Metal-Organic Framework Materials as Catalysts. Chem. Soc. Rev. 2009, 38 (5), 1450–1459. 10.1039/b807080f. 19384447

[ref2] Furukawa H.; Cordova K. E.; O’Keeffe M.; Yaghi O. M. The Chemistry and Applications of Metal-Organic Frameworks. Science 2013, 341 (6149), 1230444 10.1126/science.1230444. 23990564

[ref3] Rogge S. M. J.; Bavykina A.; Hajek J.; Garcia H.; Olivos-Suarez A. I.; Sepúlveda-Escribano A.; Vimont A.; Clet G.; Bazin P.; Kapteijn F.; Daturi M.; Ramos-Fernandez E. V.; Llabrés i Xamena F. X.; Van Speybroeck V.; Gascon J. Metal-Organic and Covalent Organic Frameworks as Single-Site Catalysts. Chem. Soc. Rev. 2017, 46 (11), 3134–3184. 10.1039/C7CS00033B. 28338128 PMC5708534

[ref4] Wei Y.-S.; Zhang M.; Zou R.; Xu Q. Metal-Organic Framework-Based Catalysts with Single Metal Sites. Chem. Rev. 2020, 120 (21), 12089–12174. 10.1021/acs.chemrev.9b00757. 32356657

[ref5] Stavila V.; Foster M. E.; Brown J. W.; Davis R. W.; Edgington J.; Benin A. I.; Zarkesh R. A.; Parthasarathi R.; Hoyt D. W.; Walter E. D.; Andersen A.; Washton N. M.; Lipton A. S.; Allendorf M. D. IRMOF-74(*n*)-Mg: A Novel Catalyst Series for Hydrogen Activation and Hydrogenolysis of C-O Bonds. Chem. Sci. 2019, 10 (42), 9880–9892. 10.1039/C9SC01018A. 32015812 PMC6977460

[ref6] Zhang Y.-Y.; Li J.-X.; Ding L.-L.; Liu L.; Wang S.-M.; Han Z.-B. Palladium Nanoparticles Encapsulated in the MIL-101-Catalyzed One-Pot Reaction of Alcohol Oxidation and Aldimine Condensation. Inorg. Chem. 2018, 57 (21), 13586–13593. 10.1021/acs.inorgchem.8b02206. 30335373

[ref7] Fan Y.; Li X.; Gao K.; Liu Y.; Meng X.; Wu J.; Hou H. Co(II)-Cluster-Based Metal-Organic Frameworks as Efficient Heterogeneous Catalysts for Selective Oxidation of Arylalkanes. CrystEngComm 2019, 21 (10), 1666–1673. 10.1039/C8CE02151A.

[ref8] Liu J.; Goetjen T. A.; Wang Q.; Knapp J. G.; Wasson M. C.; Yang Y.; Syed Z. H.; Delferro M.; Notestein J. M.; Farha O. K.; Hupp J. T. MOF-Enabled Confinement and Related Effects for Chemical Catalyst Presentation and Utilization. Chem. Soc. Rev. 2022, 51 (3), 1045–1097. 10.1039/D1CS00968K. 35005751

[ref9] Hackler R. A.; Pandharkar R.; Ferrandon M. S.; Kim I. S.; Vermeulen N. A.; Gallington L. C.; Chapman K. W.; Farha O. K.; Cramer C. J.; Sauer J.; Gagliardi L.; Martinson A. B. F.; Delferro M. Isomerization and Selective Hydrogenation of Propyne: Screening of Metal-Organic Frameworks Modified by Atomic Layer Deposition. J. Am. Chem. Soc. 2020, 142 (48), 20380–20389. 10.1021/jacs.0c08641. 33201702

[ref10] Li Z.; Schweitzer N. M.; League A. B.; Bernales V.; Peters A. W.; Getsoian A.; Wang T. C.; Miller J. T.; Vjunov A.; Fulton J. L.; Lercher J. A.; Cramer C. J.; Gagliardi L.; Hupp J. T.; Farha O. K. Sintering-Resistant Single-Site Nickel Catalyst Supported by Metal-Organic Framework. J. Am. Chem. Soc. 2016, 138 (6), 1977–1982. 10.1021/jacs.5b12515. 26836273

[ref11] Halder A.; Lee S.; Yang B.; Pellin M. J.; Vajda S.; Li Z.; Yang Y.; Farha O. K.; Hupp J. T. Structural Reversibility of Cu Doped NU-1000 MOFs under Hydrogenation Conditions. J. Chem. Phys. 2020, 152 (8), 084703 10.1063/1.5130600. 32113354

[ref12] Rimoldi M.; Bernales V.; Borycz J.; Vjunov A.; Gallington L. C.; Platero-Prats A. E.; Kim I. S.; Fulton J. L.; Martinson A. B. F.; Lercher J. A.; Chapman K. W.; Cramer C. J.; Gagliardi L.; Hupp J. T.; Farha O. K. Atomic Layer Deposition in a Metal-Organic Framework: Synthesis, Characterization, and Performance of a Solid Acid. Chem. Mater. 2017, 29 (3), 1058–1068. 10.1021/acs.chemmater.6b03880.

[ref13] Witzke R. J.; Chapovetsky A.; Conley M. P.; Kaphan D. M.; Delferro M. Nontraditional Catalyst Supports in Surface Organometallic Chemistry. ACS Catal. 2020, 10 (20), 11822–11840. 10.1021/acscatal.0c03350.

[ref14] Planas N.; Mondloch J. E.; Tussupbayev S.; Borycz J.; Gagliardi L.; Hupp J. T.; Farha O. K.; Cramer C. J. Defining the Proton Topology of the Zr_6_-Based Metal-Organic Framework NU-1000. J. Phys. Chem. Lett. 2014, 5 (21), 3716–3723. 10.1021/jz501899j. 26278741

[ref15] Wang X.; Zhang X.; Pandharkar R.; Lyu J.; Ray D.; Yang Y.; Kato S.; Liu J.; Wasson M. C.; Islamoglu T.; Li Z.; Hupp J. T.; Cramer C. J.; Gagliardi L.; Farha O. K. Insights into the Structure-Activity Relationships in Metal-Organic Framework-Supported Nickel Catalysts for Ethylene Hydrogenation. ACS Catal. 2020, 10 (16), 8995–9005. 10.1021/acscatal.0c01844.

[ref16] Liu J.; Ye J.; Li Z.; Otake K.; Liao Y.; Peters A. W.; Noh H.; Truhlar D. G.; Gagliardi L.; Cramer C. J.; Farha O. K.; Hupp J. T. Beyond the Active Site: Tuning the Activity and Selectivity of a Metal-Organic Framework-Supported Ni Catalyst for Ethylene Dimerization. J. Am. Chem. Soc. 2018, 140 (36), 11174–11178. 10.1021/jacs.8b06006. 30141922

[ref17] Goetjen T. A.; Zhang X.; Liu J.; Hupp J. T.; Farha O. K. Metal-Organic Framework Supported Single Site Chromium(III) Catalyst for Ethylene Oligomerization at Low Pressure and Temperature. ACS Sustainable Chem. Eng. 2019, 7 (2), 2553–2557. 10.1021/acssuschemeng.8b05524.

[ref18] Bernales V.; League A. B.; Li Z.; Schweitzer N. M.; Peters A. W.; Carlson R. K.; Hupp J. T.; Cramer C. J.; Farha O. K.; Gagliardi L. Computationally Guided Discovery of a Catalytic Cobalt-Decorated Metal-Organic Framework for Ethylene Dimerization. J. Phys. Chem. C 2016, 120 (41), 23576–23583. 10.1021/acs.jpcc.6b07362.

[ref19] Li Z.; Peters A. W.; Bernales V.; Ortuño M. A.; Schweitzer N. M.; DeStefano M. R.; Gallington L. C.; Platero-Prats A. E.; Chapman K. W.; Cramer C. J.; Gagliardi L.; Hupp J. T.; Farha O. K. Metal-Organic Framework Supported Cobalt Catalysts for the Oxidative Dehydrogenation of Propane at Low Temperature. ACS Cent. Sci. 2017, 3 (1), 31–38. 10.1021/acscentsci.6b00290. 28149950 PMC5269659

[ref20] Ahn S.; Nauert S. L.; Hicks K. E.; Ardagh M. A.; Schweitzer N. M.; Farha O. K.; Notestein J. M. Demonstrating the Critical Role of Solvation in Supported Ti and Nb Epoxidation Catalysts via Vapor-Phase Kinetics. ACS Catal. 2020, 10 (4), 2817–2825. 10.1021/acscatal.9b04906.

[ref21] Platero-Prats A. E.; Li Z.; Gallington L. C.; Peters A. W.; Hupp J. T.; Farha O. K.; Chapman K. W. Addressing the Characterisation Challenge to Understand Catalysis in MOFs: The Case of Nanoscale Cu Supported in NU-1000. Faraday Discuss. 2017, 201, 337–350. 10.1039/C7FD00110J. 28640304

[ref22] Bernales V.; Ortuño M. A.; Truhlar D. G.; Cramer C. J.; Gagliardi L. Computational Design of Functionalized Metal-Organic Framework Nodes for Catalysis. ACS Cent. Sci. 2018, 4 (1), 5–19. 10.1021/acscentsci.7b00500. 29392172 PMC5785762

[ref23] Wang Q.; Astruc D. State of the Art and Prospects in Metal-Organic Framework (MOF)-Based and MOF-Derived Nanocatalysis. Chem. Rev. 2020, 120 (2), 1438–1511. 10.1021/acs.chemrev.9b00223. 31246430

[ref24] Redfern L. R.; Lo W.-S.; Dillingham I. J.; Eatman J. G.; Mian M. R.; Tsung C.-K.; Farha O. K. Enhancing Four-Carbon Olefin Production from Acetylene over Copper Nanoparticles in Metal-Organic Frameworks. ACS Appl. Mater. Interfaces 2020, 12 (28), 31496–31502. 10.1021/acsami.0c08244. 32543827

[ref25] Redfern L. R.; Li Z.; Zhang X.; Farha O. K. Highly Selective Acetylene Semihydrogenation Catalyzed by Cu Nanoparticles Supported in a Metal-Organic Framework. ACS Appl. Nano Mater. 2018, 1 (9), 4413–4417. 10.1021/acsanm.8b01397.

[ref26] Ye J.; Cramer C. J.; Truhlar D. G. Organic Linker Effect on the Growth and Diffusion of Cu Clusters in a Metal-Organic Framework. J. Phys. Chem. C 2018, 122 (47), 26987–26997. 10.1021/acs.jpcc.8b09178.

[ref27] Yang Y.; Zhang X.; Kanchanakungwankul S.; Lu Z.; Noh H.; Syed Z. H.; Farha O. K.; Truhlar D. G.; Hupp J. T. Unexpected “Spontaneous” Evolution of Catalytic, MOF-Supported Single Cu(II) Cations to Catalytic, MOF-Supported Cu(0) Nanoparticles. J. Am. Chem. Soc. 2020, 142 (50), 21169–21177. 10.1021/jacs.0c10367. 33269913

[ref28] Bauer S.; Serre C.; Devic T.; Horcajada P.; Marrot J.; Férey G.; Stock N. High-Throughput Assisted Rationalization of the Formation of Metal Organic Frameworks in the Iron(III) Aminoterephthalate Solvothermal System. Inorg. Chem. 2008, 47 (17), 7568–7576. 10.1021/ic800538r. 18681423

[ref29] Han S.; Huang Y.; Watanabe T.; Dai Y.; Walton K. S.; Nair S.; Sholl D. S.; Meredith J. C. High-Throughput Screening of Metal-Organic Frameworks for CO_2_ Separation. ACS Comb. Sci. 2012, 14 (4), 263–267. 10.1021/co3000192. 22432503

[ref30] Palomba J. M.; Credille C. V.; Kalaj M.; DeCoste J. B.; Peterson G. W.; Tovar T. M.; Cohen S. M. High-Throughput Screening of Solid-State Catalysts for Nerve Agent Degradation. Chem. Commun. 2018, 54 (45), 5768–5771. 10.1039/C8CC03255F. 29781002

[ref31] Palomba J. M.; Harvey S. P.; Kalaj M.; Pimentel B. R.; DeCoste J. B.; Peterson G. W.; Cohen S. M. High-Throughput Screening of MOFs for Breakdown of V-Series Nerve Agents. ACS Appl. Mater. Interfaces 2020, 12 (13), 14672–14677. 10.1021/acsami.9b21693. 31961131

[ref32] Islamoglu T.; Otake K.; Li P.; Buru C. T.; Peters A. W.; Akpinar I.; Garibay S. J.; Farha O. K. Revisiting the Structural Homogeneity of NU-1000, a Zr-Based Metal-Organic Framework. CrystEngComm 2018, 20 (39), 5913–5918. 10.1039/C8CE00455B.

[ref33] Bu J.; Liu Z.; Ma W.; Zhang L.; Wang T.; Zhang H.; Zhang Q.; Feng X.; Zhang J. Selective Electrocatalytic Semihydrogenation of Acetylene Impurities for the Production of Polymer-Grade Ethylene. Nat. Catal. 2021, 4 (7), 557–564. 10.1038/s41929-021-00641-x.

[ref34] Chai Y.; Han X.; Li W.; Liu S.; Yao S.; Wang C.; Shi W.; da-Silva I.; Manuel P.; Cheng Y.; Daemen L. D.; Ramirez-Cuesta A. J.; Tang C. C.; Jiang L.; Yang S.; Guan N.; Li L. Control of Zeolite Pore Interior for Chemoselective Alkyne/Olefin Separations. Science 2020, 368 (6494), 1002–1006. 10.1126/science.aay8447. 32467390

[ref35] Oakton E.; Vilé G.; Levine D. S.; Zocher E.; Baudouin D.; Pérez-Ramírez J.; Copéret C. Silver Nanoparticles Supported on Passivated Silica: Preparation and Catalytic Performance in Alkyne Semi-Hydrogenation. Dalton Trans. 2014, 43 (40), 15138–15142. 10.1039/C4DT01320D. 25178410

[ref36] Bridier B.; López N.; Pérez-Ramírez J. Partial Hydrogenation of Propyne over Copper-Based Catalysts and Comparison with Nickel-Based Analogues. J. Catal. 2010, 269 (1), 80–92. 10.1016/j.jcat.2009.10.019.

[ref37] Ossipoff N. J.; Cant N. W. The Hydrogenation and Oligomerization of Propyne over an Ion-Exchanged Copper on Silica Catalyst. J. Catal. 1994, 148 (1), 125–133. 10.1006/jcat.1994.1193.

[ref38] Wehrli J. T.; Thomas D. J.; Wainwright M. S.; Trimm D. L.; Cant N. W. Selective Hydrogenation of Propyne over Supported Copper Catalysts: Influence of Support. Appl. Catal. 1991, 70 (1), 253–262. 10.1016/S0166-9834(00)84168-8.

[ref39] Howarth A. J.; Liu Y.; Li P.; Li Z.; Wang T. C.; Hupp J. T.; Farha O. K. Chemical, Thermal and Mechanical Stabilities of Metal-Organic Frameworks. Nat. Rev. Mater. 2016, 1 (3), 15018 10.1038/natrevmats.2015.18.

[ref40] Shahriari B.; Swersky K.; Wang Z.; Adams R. P.; de Freitas N. Taking the Human Out of the Loop: A Review of Bayesian Optimization. Proceedings of the IEEE 2016, 104 (1), 148–175. 10.1109/JPROC.2015.2494218.

[ref41] Shields B. J.; Stevens J.; Li J.; Parasram M.; Damani F.; Alvarado J. I. M.; Janey J. M.; Adams R. P.; Doyle A. G. Bayesian Reaction Optimization as a Tool for Chemical Synthesis. Nature 2021, 590 (7844), 89–96. 10.1038/s41586-021-03213-y. 33536653

[ref42] Naito Y.; Kondo M.; Nakamura Y.; Shida N.; Ishikawa K.; Washio T.; Takizawa S.; Atobe M. Bayesian Optimization with Constraint on Passed Charge for Multiparameter Screening of Electrochemical Reductive Carboxylation in a Flow Microreactor. Chem. Commun. 2022, 58 (24), 3893–3896. 10.1039/D2CC00124A. 35226032

[ref43] Häse F.; Roch L. M.; Kreisbeck C.; Aspuru-Guzik A. Phoenics: A Bayesian Optimizer for Chemistry. ACS Cent. Sci. 2018, 4 (9), 1134–1145. 10.1021/acscentsci.8b00307. 30276246 PMC6161047

[ref44] Isbrandt E. S.; Sullivan R. J.; Newman S. G. High Throughput Strategies for the Discovery and Optimization of Catalytic Reactions. Angew. Chem., Int. Ed. 2019, 58 (22), 7180–7191. 10.1002/anie.201812534. 30576045

[ref45] Langner S.; Häse F.; Perea J. D.; Stubhan T.; Hauch J.; Roch L. M.; Heumueller T.; Aspuru-Guzik A.; Brabec C. J. Beyond Ternary OPV: High-Throughput Experimentation and Self-Driving Laboratories Optimize Multi-Component Systems. Adv. Mater. 2020, 32, 1907801 10.1002/adma.201907801. 32049386

[ref46] Vilé G.; Baudouin D.; Remediakis I. N.; Copéret C.; López N.; Pérez-Ramírez J. Silver Nanoparticles for Olefin Production: New Insights into the Mechanistic Description of Propyne Hydrogenation. ChemCatChem. 2013, 5 (12), 3750–3759. 10.1002/cctc.201300569.

[ref47] Ward L.; Agrawal A.; Choudhary A.; Wolverton C. A General-Purpose Machine Learning Framework for Predicting Properties of Inorganic Materials. npj Comput. Mater. 2016, 2 (1), 16028 10.1038/npjcompumats.2016.28.

[ref48] McCullough K.; Williams T.; Mingle K.; Jamshidi P.; Lauterbach J. High-Throughput Experimentation Meets Artificial Intelligence: A New Pathway to Catalyst Discovery. Phys. Chem. Chem. Phys. 2020, 22 (20), 11174–11196. 10.1039/D0CP00972E. 32393932

[ref49] Zheng J.; Ye J.; Ortuño M. A.; Fulton J. L.; Gutiérrez O. Y.; Camaioni D. M.; Motkuri R. K.; Li Z.; Webber T. E.; Mehdi B. L.; Browning N. D.; Penn R. L.; Farha O. K.; Hupp J. T.; Truhlar D. G.; Cramer C. J.; Lercher J. A. Selective Methane Oxidation to Methanol on Cu-Oxo Dimers Stabilized by Zirconia Nodes of an NU-1000 Metal-Organic Framework. J. Am. Chem. Soc. 2019, 141 (23), 9292–9304. 10.1021/jacs.9b02902. 31117650

[ref50] Mian M. R.; Redfern L. R.; Pratik S. M.; Ray D.; Liu J.; Idrees K. B.; Islamoglu T.; Gagliardi L.; Farha O. K. Precise Control of Cu Nanoparticle Size and Catalytic Activity through Pore Templating in Zr Metal-Organic Frameworks. Chem. Mater. 2020, 32 (7), 3078–3086. 10.1021/acs.chemmater.0c00059.

[ref51] Ye J.; Gagliardi L.; Cramer C. J.; Truhlar D. G. Computational Screening of MOF-Supported Transition Metal Catalysts for Activity and Selectivity in Ethylene Dimerization. J. Catal. 2018, 360, 160–167. 10.1016/j.jcat.2017.12.007.

[ref52] Frisch M. J.; Trucks G. W.; Schlegel H. B.; Scuseria G. E.; Robb M. A.; Cheeseman J. R.; Scalmani G.; Barone V.; Petersson G. A.; Nakatsuji H.; Li X.; Caricato M.; Marenich A. V.; Blolino J.; Janesko B. G.; Gomperts R.; Mennucci B.; Hratchian H. P.; Ortiz J. V.; Izmaylov A. F.; Sonnenberg J. L.; Williams-Young D.; Ding F.; Lipparini F.; Egidi F.; Goings J.; Peng B.; Petrone A.; Henderson T.; Ranasinghe D.; Zakrzewski V. G.; Gao J.; Rega N.; Zheng G.; Liang W.; Hada M.; Ehara M.; Toyota K.; Fukuda R.; Hasegawa J.; Ishida M.; Nakajima T.; Honda Y.; Kitao O.; Nakai H.; Vreven T.; Throssell K.; Montgomery J. A. Jr.; Peralta J. E.; Oligaro F.; Bearpark M. J.; Heyd J. J; Brothers E. N.; Kudin K. N.; Staroverov V. N.; Keith T. A.; Kobayashi R.; Normand J.; Ragavachari K.; Rendell A. P.; Burant J. C.; Iyengar S. S.; Tomasi J.; Cossi M.; Millam J. M.; Klene M.; Adamo C.; Cammi R.; Ochterski J. W.; Martin R. L.; Morokuma K.; Farkas O.; Foresman J. B.; Fox D. J. Gaussian 16; Gaussian, Inc.: Wallingford, CT, 2016.

[ref53] Zhao Y.; Truhlar D. G. The M06 Suite of Density Functionals for Main Group Thermochemistry, Thermochemical Kinetics, Noncovalent Interactions, Excited States, and Transition Elements: Two New Functionals and Systematic Testing of Four M06-Class Functionals and 12 Other Functionals. Theor. Chem. Acc. 2008, 120 (1), 215–241. 10.1007/s00214-007-0310-x.

[ref54] Weigend F.; Ahlrichs R. Balanced Basis Sets of Split Valence, Triple Zeta Valence and Quadruple Zeta Valence Quality for H to Rn: Design and Assessment of Accuracy. Phys. Chem. Chem. Phys. 2005, 7 (18), 3297–3305. 10.1039/b508541a. 16240044

[ref55] Ribeiro R. F.; Marenich A. V.; Cramer C. J.; Truhlar D. G. Use of Solution-Phase Vibrational Frequencies in Continuum Models for the Free Energy of Solvation. J. Phys. Chem. B 2011, 115 (49), 14556–14562. 10.1021/jp205508z. 21875126

[ref56] Ortuño M. A.; Bernales V.; Gagliardi L.; Cramer C. J. Computational Study of First-Row Transition Metals Supported on MOF NU-1000 for Catalytic Acceptorless Alcohol Dehydrogenation. J. Phys. Chem. C 2016, 120 (43), 24697–24705. 10.1021/acs.jpcc.6b06381.

[ref57] Kresse G.; Furthmüller J. Efficient Iterative Schemes for Ab Initio Total-Energy Calculations Using a Plane-Wave Basis Set. Phys. Rev. B 1996, 54 (16), 11169–11186. 10.1103/PhysRevB.54.11169. 9984901

[ref58] Kresse G.; Furthmüller J. Efficiency of Ab-Initio Total Energy Calculations for Metals and Semiconductors Using a Plane-Wave Basis Set. Comput. Mater. Sci. 1996, 6 (1), 15–50. 10.1016/0927-0256(96)00008-0.

[ref59] Perdew J. P.; Burke K.; Ernzerhof M. Generalized Gradient Approximation Made Simple. Phys. Rev. Lett. 1996, 77 (18), 3865–3868. 10.1103/PhysRevLett.77.3865. 10062328

[ref60] Grimme S.; Antony J.; Ehrlich S.; Krieg H. A Consistent and Accurate Ab Initio Parametrization of Density Functional Dispersion Correction (DFT-D) for the 94 Elements H-Pu. J. Chem. Phys. 2010, 132 (15), 154104 10.1063/1.3382344. 20423165

[ref61] Henkelman G.; Jónsson H. Improved Tangent Estimate in the Nudged Elastic Band Method for Finding Minimum Energy Paths and Saddle Points. J. Chem. Phys. 2000, 113 (22), 9978–9985. 10.1063/1.1323224.

