# Supplementary material for: High-Throughput Experimentation, Theoretical Modeling, and Human Intuition: Lessons Learned in Metal–Organic-Framework-Supported Catalyst Design
Source: ACS Cent Sci. 2023 Jan 26;9(2):266–76. doi: 10.1021/acscentsci.2c01422 (PMC9951283; doi:10.1021/acscentsci.2c01422)
Supplement: Supplementary file 1 — oc2c01422_si_001.pdf [file oc2c01422_si_001.pdf]

Supporting Information

High-Throughput Experimentation, Theoretical Modeling, and Human Intuition: Lessons Learned in Metal-Organic Framework-Supported Catalyst Design

Katherine E. McCullough,[†,#] Daniel S. King,[‡,#] Saumil P. Chheda,[¶] Magali S. Ferrandon,[†] Timothy A. Goetjen,[‖,†] Zoha H. Syed, [‖,†] Trent R. Graham[⅃], Nancy M. Washton[⅃], Omar K. Farha,[‖] Laura Gagliardi,*[,‡,§,⊥] and Massimiliano Delferro *[†,§]

†*Chemical Sciences and Engineering Division, Argonne National Laboratory, Lemont, IL 60439 USA*

‡*Department of Chemistry, University of Chicago, Chicago, IL 60637 USA*

¶*Department of Chemical Engineering and Materials Science, University of Minnesota, Minneapolis, MN 55455 USA*

§*Pritzker School of Molecular Engineering, University of Chicago, Chicago, IL 60637 USA*

[⅃]*Pacific Northwest National Laboratory, Richland, WA 99354 USA*

‖*Department of Chemistry, Northwestern University, Evanston, IL 60208 USA*

⊥ *James Franck Institute, Chicago Center for Theoretical Chemistry, University of Chicago, Chicago, IL 60637 USA*

#*Contributed equally to this work*

E-mail: lgagliardi@uchicago.edu, delferro@anl.gov

Table of Content

**1. Experimental Selection Methodology**	S2
**2. ICP Weight Loadings**	S16
**3. Mass Balance**	S17
**4. Powder X-ray Diffraction of Bare NU-1000 vs. Cu/NU-1000**	S18
**5. Stability Test**	S19
**6. Raman Spectroscopy**	S20
**7. DFT Energy Profile**	S21
**8. References**	S25

1. Experimental Selection Methodology. This section outlines the machine learning methodologies used in this work to accelerate the discovery of catalytic materials.

Campaign S1. The selection algorithm employed in campaign 1 utilized a separate Bayesian optimization scheme for each metal investigated. This optimization algorithm models the objective function with Gaussian process regression and selects new experiments via an acquisition function,[1] which balances objective optimization and exploration of the parameter space. The use of this approach for optimizing experimental parameters is well established[2–6] and makes minimal assumptions about the objective function while efficiently exploring the parameter space. Bayesian optimization was used via the GPyOPT[7] with the standard expected improvement acquisition function. Bayesian optimization was employed for each metal in discrete ranges over the following variables in GPyOpt:

**Variable Name**	**Value Range**
Metal Loading (wt %)	0.5 – 5
Total Flow (sccm)	2.5 – 20
H_2 (vol%)	0 – 5
Reaction Temperature (°C)	100 – 250
H_2 Reduction Temperature (°C)	150 – 225
Air Reduction Temperature (°C)	125 – 225

During each round of experiments, suggestions were made individually for all 23 metals and prioritized for execution based on previous literature results and metal. The expected improvement acquisition function was used to suggest experiments for all metals.

B

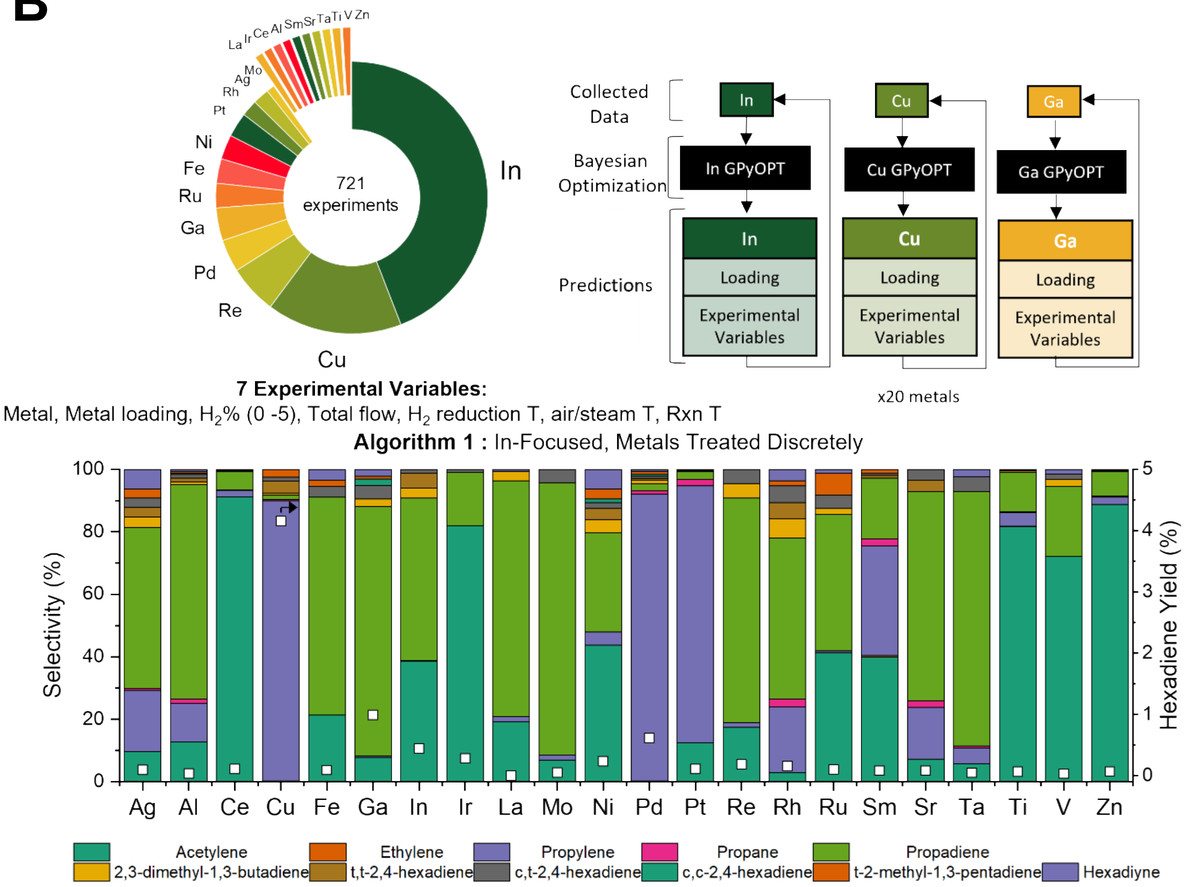

Figure S1: Summary of initial campaign to optimize hexadiene yield over 22 different catalytically active metals. The distribution of the metals experimentally tested is shown in the pie chart (expanded for clarity) and totaled to 721 experiments. Individual Bayesian optimization schemes were created for each metal using GPyOPT utilizing the collected data to make predictions, where data from each iteration were added to the algorithm to make the next set of predictions. The bottom graph indicates the highest hexadiene yield (white squares) for each metal and the associated selectivity distribution.

Campaign S2. Campaign 2 began with an initial screening of all metals at the following parameters:

Variable Name	Value
Metal Loading (wt%)	1
Space Velocity (mL/min/g_{ca})	2000
H_2 (vol%)	40
Reaction Temperature (°C)	100, 150, 200
H_2 Reduction Temperature (°C)	200
Air Reduction Temperature (°C)	200

Based on the success of Cu from the campaign 1 results and its observed dependence on metal loading, Cu loadings of 4% and 0.5% were also tested in the initial screening. This screening resulted in Cu yields of 15.4% (at the 1% metal loading and reaction temperature 200 °C, which was much larger than the second-highest hexadiene-yielding metal of Zn (0.26% yield). Due to this drastic outperformance of Cu, it was decided that our selection algorithm should focus on Cu. However, since only one set of operating conditions can be tested for Cu at a time, it also made sense to use the additional reactor capacity to investigate the performance of other metals.

The experimental selection algorithm in campaign 2 was hence redesigned to suit these requirements, consisting of a dual-model approach in which Model 1 would select operating conditions for Cu and Model 2 would attempt to interpolate between metals to select alternative metals and metal loadings to screen at the selected operating conditions. To avoid overly biasing the model towards the low-performing conditions of the initial campaign, previously collected Cu data from the initial campaign was not used. Selectivities and conversions were averaged over identical trials if present. Finally, Co was added to the search space at the beginning of campaign 2, bringing the total metal count to 23.

Model S1. The following experimental parameters were selected for the screening of Cu operating conditions in campaign 2:

Variable Name	Value Range
Metal Loading (wt%)	0.5 – 5
Space Velocity (mL/min/g_{cat})	50 – 5000
H_2 (vol%)	0 – 80
Reaction Temperature (°C)	100 – 250

In contrast to campaign 1, the hydrogen reduction temperature and air reduction temperature were both fixed to 200 °C as they were not observed to significantly change experimental yields. Thus, the task of model 1 was to effectively explore this range of four experimental variables.

While this would seem to be a task well-suited for the Bayesian optimization tools utilized in campaign 1, we disliked that Bayesian optimization did not seem appropriately biased towards experimental intuition, often suggesting edge conditions that seemed unlikely to give good results (stemming from the uniform prior Bayesian optimization places on the entire parameter space). To achieve this aim, we developed a very simple model which projects the currently collected experimental data onto its principal component analysis (PCA) eigenvectors and uses a K-nearest-neighbors (KNN) model with K = 3 to select new experiments within this space. The scheme is outlined as follows:

- [If no data exists] Collect an initial set of N_{expt} experiments following experimental intuition about where the highest yield will lie.

- Gather the collected experimental data into an $N_{expt} \times N_{var}$ table X in which the columns index the four exploratory variables N_{var}: loading, H_2 %, reaction temperature, and space velocity.

- Normalize the columns via min-max normalization using the set parameter ranges on the exploratory variables to form the normalized data matrix U:

$$U_{ij} = \frac{X_{ij}}{max_j - min_j}$$

  where max_j and min_j are the upper and lower ranges on the exploratory variables (in the table above).

- Center the data by subtracting the mean of each column:

$$U_{ij} = U_{ij} - \frac{1}{N_{expt}} \sum_i U_{ij}$$

- Find the PCA eigenvectors and explained variances of the data via diagonalization of the covariance matrix $U^T U$. Keep enough eigenvectors v (forming the linear subspace V) such that the total explained variance is greater than 0.95.

- Split the 4 parameters into L segments of equal length to form a L × L × L × L search space of L^4 points over the exploratory variables. Min-max normalize these points using the

upper and lower limits (as done for U), and then project this search space onto the v PCA eigenvectors from the previous step. Here we set $L = 10$.

- Using the maximum and minimum values of the projected data on each PCA eigenvector, form a v-dimensional $N \times N \times N$... search hypercube Z in V spanning each range of the projected search space along the v PCA eigenvectors in N segments of equal length. Here we use $N = 60$.

- Project the normalized experimental data U onto the v PCA eigenvectors, and collect associated target data (e.g., total hexadiene yield) y. Use a K-Nearest-Neighbors (KNN) model to predict y in PCA space, using uniform weighting with the number of nearest neighbors set to 3.

- Using the trained KNN model, make predictions on the target variable for all points in the search hypercube Z and find the point in Z for which the predicted yield is highest, q. If there are multiple points, average all predicted highest-yield points to find q (i.e., take the centroid)

- Transform q back to the dimensions of exploratory variables by expanding its PCA eigenvector decomposition and re-scaling to the appropriate dimensions (undoing the minmax normalization).

- Return the proposed experiment q

The PCA and K-Nearest-Neighbors algorithms used above were utilized following their implementations in sklearn.

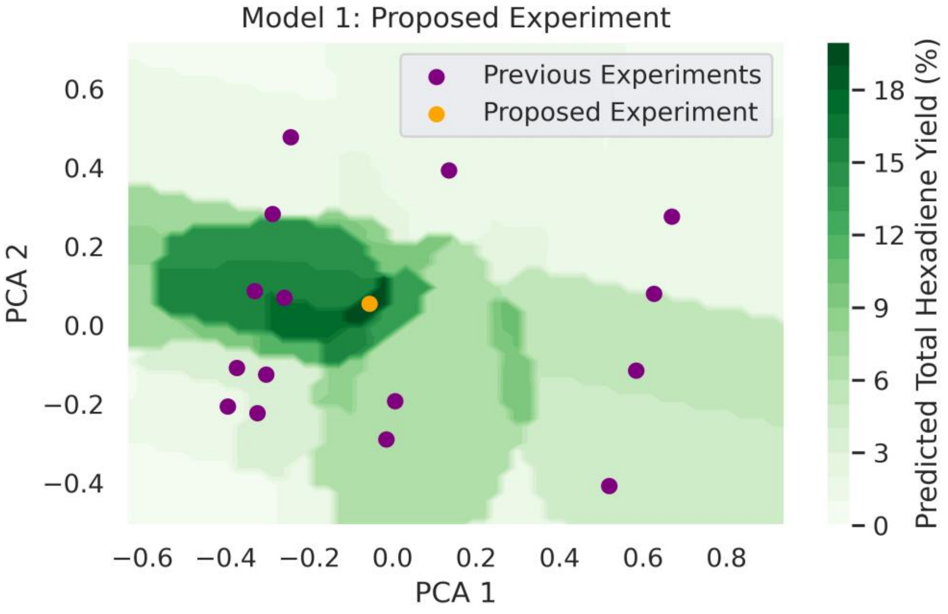

Figure S2. Example of a proposed experiment using the PCA-KNN methodology described above. The collected experimental data is plotted on top of a contour plot of KNN predictions in the search grid *Z*, with both projected onto the PCA eigenvectors with maximum explained variance (the KNN predictions shown are the maximum predicted yields in *Z* having the specified values in the first two PCA eigenvectors).

Despite the simplicity of the KNN model for predicting yields in PCA space, we find it to have an excellent mean absolute error of only 2% yield in leave-one-out cross-validation on the final set of Cu data, which compares well to the 1.6% mean absolute error when using the much more sophisticated XGBoost algorithm, and well-within the standard deviation of 4.5% yield. However, this model could easily be substituted with a standard Bayesian optimizer if so desired.

Model S2. Given the experimental operating conditions for Cu suggested by model 1, model 2 uses a series of metal features in combination with the suggested operating conditions to screen different metals and loadings to try simultaneously. Model 2 consists of an XGBoost regression model trained on the current set of experimental data (including from campaign 1, but excluding Cu from campaign 1) which is used to screen a grid of the 22 non-Cu metals at 5 different loadings to try in tandem with the Cu experiment suggested by model 1.

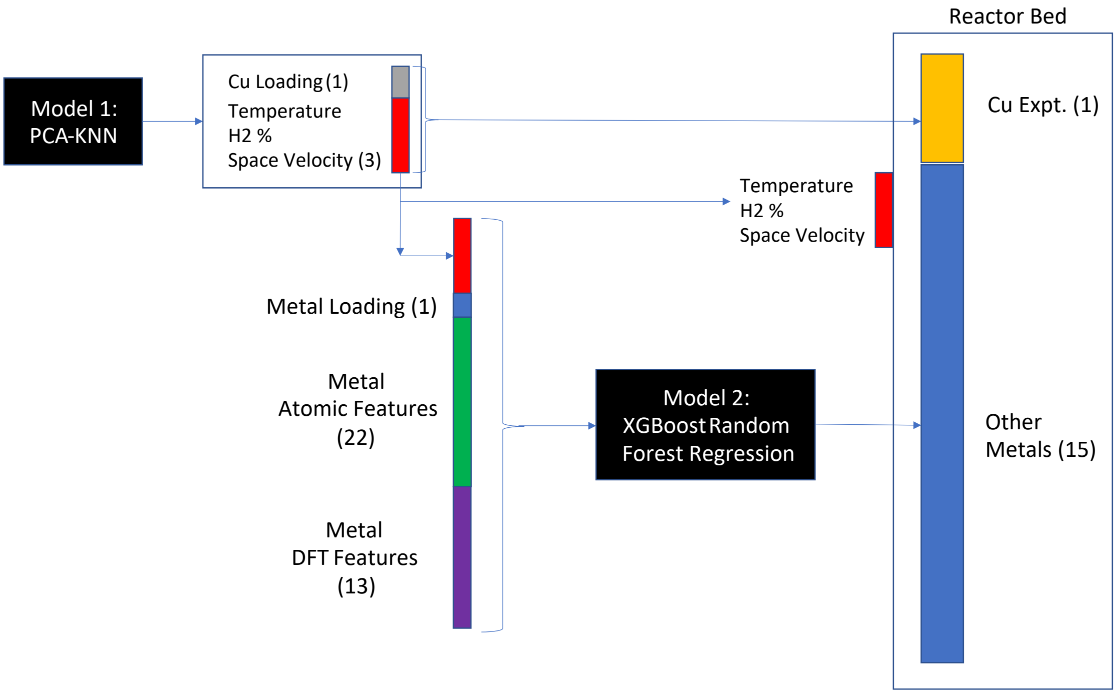

Figure S3. Diagram of how model 2 is constructed and interacts with model 1, complementary to Figure 2 in the main text. Model 1 provides a set of experimental conditions (space velocity, temperature, and H_2%) which are used by model 2 to predict complementary metals and metal loadings to test at those conditions.

The following features are used by model 2 to predict yields for a given metal and metal loading:

Operating Conditions from Model 1 (3). Reaction temperature, H_2 %, Space Velocity

Metal Loading (1). 0-5 %

Atomic Features (22). We have used 22 different elemental properties of each metal used, including the electronegativity, atomic number, column, row, and number of valence electrons of different types as put forward by Ward and coworkers.[8] These features are well-known and have been applied in similar projects.[9] The features are listed here as follows: Atomic Number, Mendeleev Number, Atomic Weight, Melting Temperature, Column, Row, Covalent Radius, Electronegativity, NsValence, NpValence, NdValence, NfValence, NValence, NUnfilled, NpUnfilled, NdUnfilled, NfUnfilled, NUnfilled, GSvolume_pa, GSbandgap, GSmagmom, and SpaceGroupNumber.

DFT Features (13). In addition to publicly available atomic features, we have enhanced model 2 using our own characterizations of the different metals using density functional theory. We have constructed cluster models of single metal centers deposited onto NU-1000 nodes and evaluated the adsorption energies of different reaction intermediates. These 3-coordinate single metal centers were chosen to generate uniform data across several different transition metals, stemming from literature precedent.[10–13]

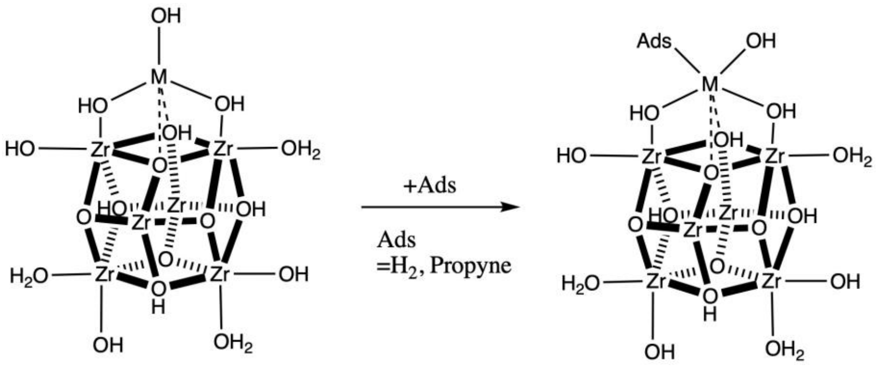

Figure S4. Schematic of adsorption energies calculated with DFT to featurize and interpolate between metals.

Figure S4 shows the adsorption energies calculated to featurize the different metals in model 2. Cluster models were optimized with use of the M06-L functional[14] using the def2-SVP[1]/24/2023 2:40:00 PM for non-metals and the def2-TZVP[1]/24/2023 2:40:00 PM basis for zirconium and deposited metal centers in Gaussian 16.[15] The SDD pseudopotential[16] was used for all metals present beyond the fourth row of the periodic table. Clusters were optimized at all reasonable spin multiplicities at all intermediates and the lowest energy multiplicity was used for each reaction profile. Adsorption energies were calculated for only 9 metals: Al, Cu, Fe, In, Ni, Pd, Pt, Rh, and Ru. However, the flexibility of the XGBoost model allows for use of these features even though data is missing for several metals.

Table S1. Features collected for the 9 different metals analyzed using DFT. M is the multiplicity (2S+1) found most stable for the computed single-metal active site with adsorbed H_2, adsorbed propyne (Prop), and nothing adsorbed (Bare). Additionally, we collected 5 thermodynamic features for each adsorption reaction: ΔE, ΔG, ΔH, TΔS, and the change in the zero-point vibrational energy ΔZPE. All values besides the multiplicities are listed in kcal/mol.

	H_2	Prop	Bare	H_2	Prop	H_2	Prop	H_2	Prop	H_2	Prop	H_2	Prop
	M	M	M	ΔE	ΔE	ΔG	ΔG	ΔH	ΔH	TΔS	TΔS	ΔZPE	ΔZPE
Al	1	1	1	-1.0	-7.3	6.9	6.5	0.3	-6.0	-6.5	-12.5	1.7	0.9
Cu	2	2	2	-2.6	-12.7	6.2	1.6	-1.2	-11.4	-7.4	-13.0	2.1	1.0
Fe	5	5	5	-1.4	-29.5	7.0	-13.8	-0.1	-28.1	-7.1	-14.3	1.9	1.8
In	1	1	1	-1.2	-6.9	7.1	8.8	0.1	-5.3	-7.1	-14.1	1.8	1.9
Ni	3	3	3	-4.3	-20.7	4.8	-5.6	-2.9	-19.7	-7.6	-14.1	2.2	1.4
Pd	1	1	1	-21.2	-35.0	-9.0	-20.3	-18.3	-33.9	-9.3	-13.6	4.5	1.2
Pt	3	3	1	-6.7	-25.6	3.9	-11.7	-4.3	-24.6	-8.2	-12.9	3.9	1.0
Rh	1	1	3	-15.4	-27.0	-1.5	-10.5	-12.2	-25.8	-10.7	-15.3	5.2	1.6
Ru	3	3	5	-22.6	-47.9	-8.8	-31.1	-19.4	-46.3	-10.6	-15.3	5.3	2.3

Table S1 shows the 5 thermodynamic features we collected for hydrogen and propyne adsorption as well as the most stable spin multiplicities (2S +1) we found for each computational single metal site with adsorbed hydrogen, propyne, and without anything adsorbed (bare). In total we collected 13 features for each of the 9 metals we computed. Thermochemical quantities were calculated using GoodVibes[17] at 318.15 K. Vibrational frequencies less than 100 cm^{-1} were rounded up to 100 cm^{-1} following the approach of Cramer and Truhlar.[18]

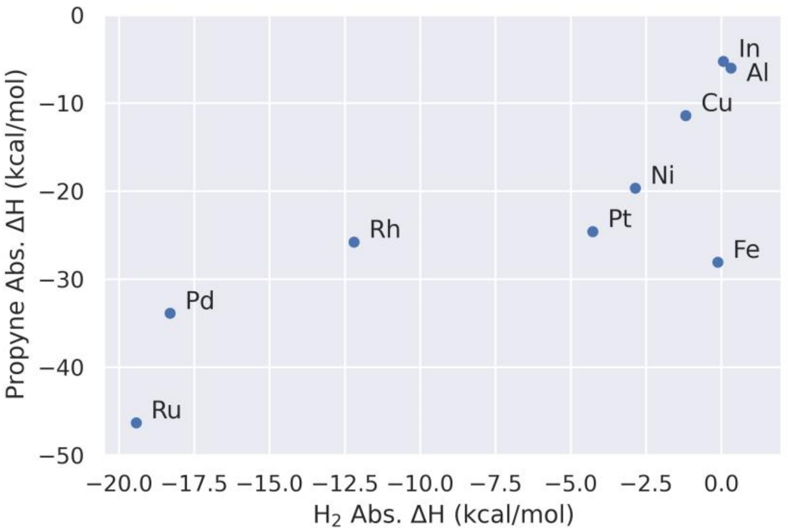

Figure S5. Scatter plot of calculated hydrogen adsorption enthalpies against propyne adsorption enthalpies.

Figure S5 shows our calculated hydrogen adsorption enthalpies against our calculated propyne adsorption energies for 9 different metals. Our results for Cu seem to indicate that catalysis is favorable if both propyne and hydrogen are weakly adsorbed.

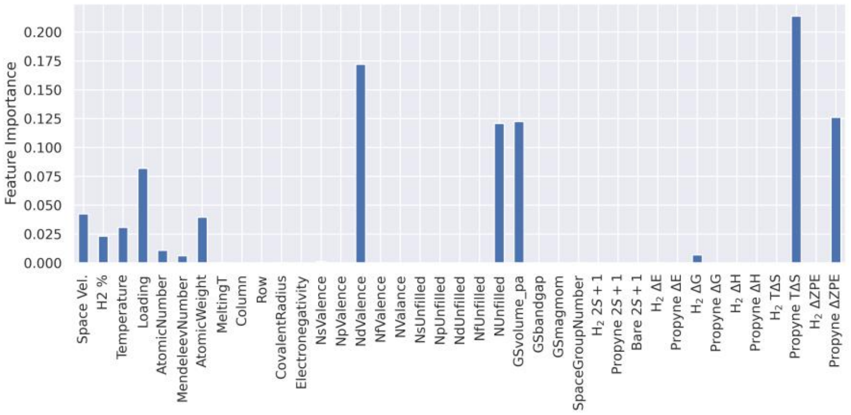

Figure S6. Feature importance of an XGBoost model trained on the final set of all 1373 experiments, excluding Cu data collected pre-1/26, and using all collected features (atomic and DFT)

Figure S6 shows the feature importance of an XGBoost regression model trained to predict metal yields from the entire set of 39 elemental and DFT-obtained features. All experimental variables (space velocity, H_2 %, temperature, and metal loading) as well as key elemental and DFT features

(Ndvalence, NUnfilled, Propyne adsorption TΔS, and Propyne adsorption ΔZPE) contribute importantly to the predictions of the model.

Model performance. Leave-one-out cross validation on the final set of Cu data yields a mean absolute error for the KNN-PCA model of 2.0%, comparable to the much more sophisticated XGBoost regression algorithm which yields a mean absolute error of 1.6% (both well-within the yield standard deviation of 4.5%). Leave-one-out cross validation of model 2 on the final set of experimental data yields a mean absolute error of 0.3% hexadiene yield (well-within the experimental standard deviation of 1.7%).

Differences in activity between Ni, Pd, and Cu. To better understand variations in these three catalysts and the effect of H_2 inlet concentration on selectivity, the metal loading (5 wt. %), space velocity (2,000 mL/min/g_{cat}) and temperature (200 °C) were held constant, and the inlet H_2 vol% was varied from 0, 4, 8, 16, 32 to 64% for Pd, Ni and Cu catalysts supported on NU-1000. The conversion and selectivity to hexadiene, propylene and propane at 200°C for these catalysts are shown in Figure S7.

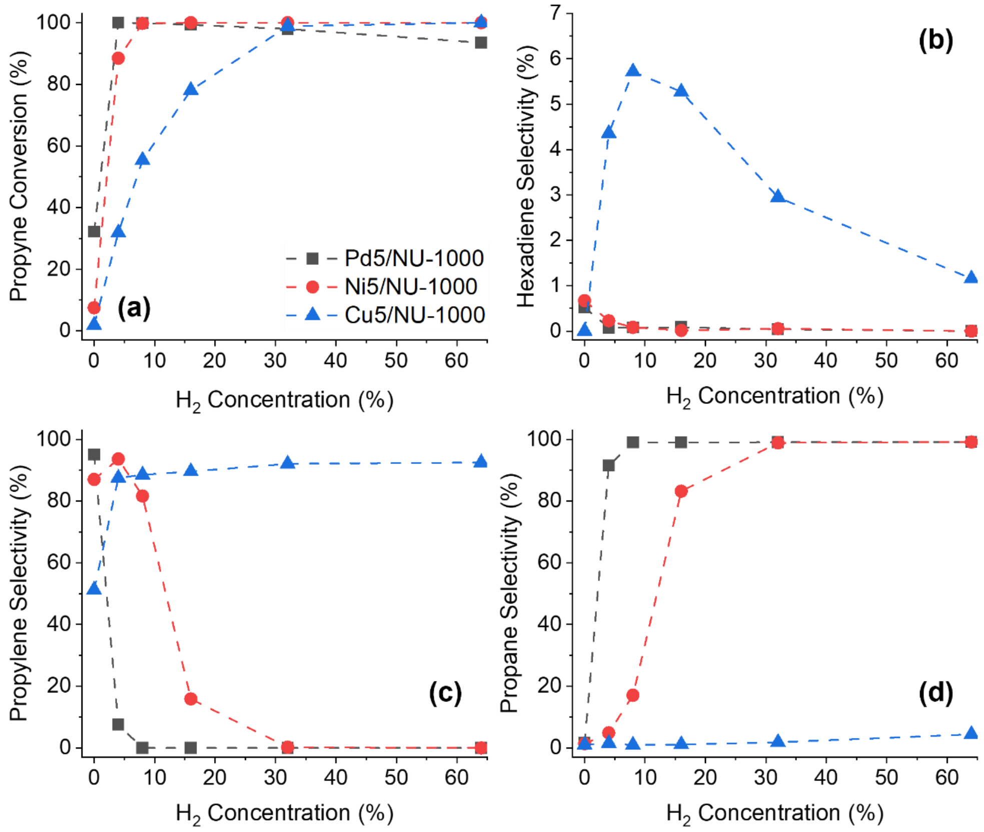

Figure S7. (a) Propyne conversion, (b) hexadiene selectivity, (c) propylene selectivity and (d) propane selectivity for Pd5/NU-1000 (black trace), Ni5/NU-1000 (red trace) and Cu5/NU-1000 (blue trace) as a function of inlet H_2 concentration. Reaction conditions: T = 200°C, 2,000 mL/min/g_{cat}, 0 – 64 vol% H_2 in balance 2% propyne/Ar, P = 1 atm.

It is readily apparent that the inlet partial pressure favoring hexadiene formation over Cu is not like those for Ni or Pd,[19–23] and that there is a strong interplay between conversion and selectivity that varies as a function of H_2 concentration. Literature suggests that alkene formation is driven by thermodynamic selectivity and hindering hydride formation.[21,24] Pd based catalysts require control over the subsurface H_2 layer, which is prone to hydrogenate ethyne to ethane, and that the selectivity to ethylene is governed by the interplay between hydride and carbide phases.[25–27] Others observe that an increase in H_2 partial pressure in the inlet results in a higher selectivity to propene and oligomers are produced at the lower H_2:C_3H_4 ratios.[28] Furthermore, differences in selectivity and yield for hexadiene shown under these conditions do not produce significant amounts of hexadiene, which showcases the interplay that exists between metal loading, reaction temperature, space velocity and H_2 concentration. The one-at-a-time approach that would be necessary to screen these variables independently quickly becomes overly time consuming.

Highest Yields in Campaign 1

Table S2. Metal loading, inlet hydrogen concentration, space velocity, reactor temperature and propyne conversion corresponding to the highest hexadiene yield for each metal studied during the initial campaign.

Metal	Loading (%)	H_2 (%)	Space Velocity (mL/min/g_{cat})	Reaction T (°C)	Propyne Conversion (%)	Total Hexadiene Yield (%)
Ag	0.5	5	2000	225	1.55	0.09
Al	3	3	2000	225	1.20	0.03
Ce	3	5	2000	250	20.49	0.11
Cu	4	5	2000	250	84.04	4.15
Fe	5	0	2000	225	2.63	0.09
Ga	3	0	2000	250	15.77	0.99
In	5	0	2000	225	7.37	0.44
Ir	5	0	2000	250	32.20	0.28
La	5	0	2000	250	2.42	0.00
Mo	3	0	2000	175	1.14	0.05
Ni	5	0	320	200	3.48	0.23
Pd	5	0	160	100	33.25	0.61
Pt	0.5	3	2000	250	31.68	0.11
Re	0.5	0	2000	225	3.94	0.18
Rh	5	3	2000	175	1.45	0.15
Ru	5	2.5	2000	200	2.33	0.10
Sm	3	3	2000	250	5.15	0.08
Sr	1	5	2000	200	1.16	0.08
Ta	3	5	2000	200	0.95	0.05
Ti	5	3	2000	250	9.78	0.06
V	5	0	2000	225	1.64	0.03
Zn	1	5	2000	200	15.50	0.07

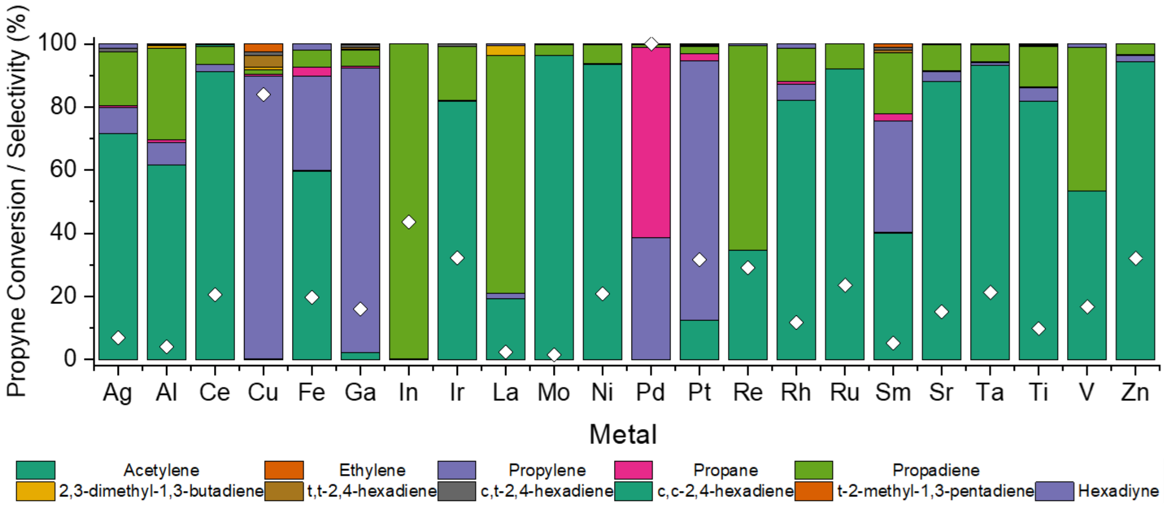

Figure S8. Product distribution for each metal corresponding to conditions that resulted in the highest propyne conversion for each metal in campaign 1. Reaction conditions and metal loading are given in the previous table

Highest Yields in Campaign 2

Table S3. Metal loading, inlet hydrogen concentration, space velocity, reactor temperature and propyne conversion corresponding to the highest hexadiene yield for each metal studied during Campaign 2 (not averaged over identical trials).

Metal	Loading (%)	H_2 (%)	Space Velocity (mL/min/g_{cat})	Reaction T (°C)	Propyne Conversion (%)	Total Hexadiene Yield (%)
Ag	5	20	562.5	250	48.42	0.58
Al	5	80	1644	200	44.59	0.19
Ce	3	80	1644	200	69.27	0.57
Co	1	40	2000	200	7.27	0.33
Cu	2	20	1000	200	99.59	25.88
Fe	1	20	1400	150	15.87	0.24
Ga	1	34	4080	200	67.92	0.56
In	1	40	2000	100	1.66	0.14
Ir	0.5	38	1020	200	9.17	0.17
La	1	40	2000	100	61.25	0.24
Mo	4	38	1020	200	2.31	0.13
Ni	5	38.5	162	175	98.93	7.03
Pd	5	13.5	460	200	99.63	7.34
Pt	1	38	1644	200	99.79	0.06
Re	1	38	1020	200	26.67	0.17
Rh	1	40	2000	100	45.83	0.13
Ru	5	30	500	200	91.12	0.87
Sm	1	40	2000	200	36.17	0.11
Sr	0.5	38	1020	200	2.39	0.11
Ta	1	40	2000	150	1.27	0.07
Ti	3	41	1725	188	7.88	0.28
V	1	80	1644	200	44.25	0.12
Zn	4	61	1736	188	2.95	0.52

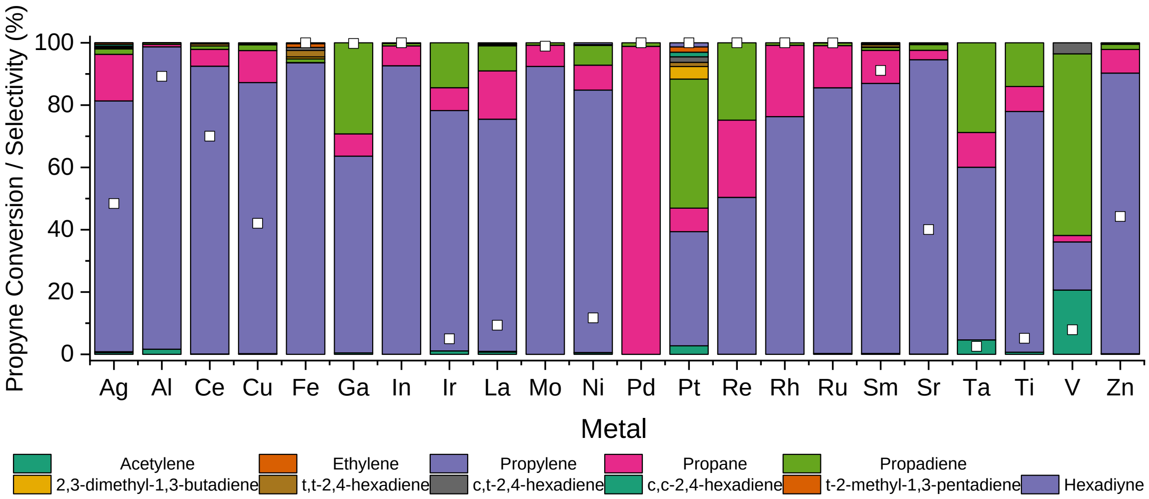

Figure S9. Product distribution for each metal corresponding to conditions that resulted in the highest propyne conversion for each metal in campaign 2.

Comparison of Campaign 1 and Campaign 2

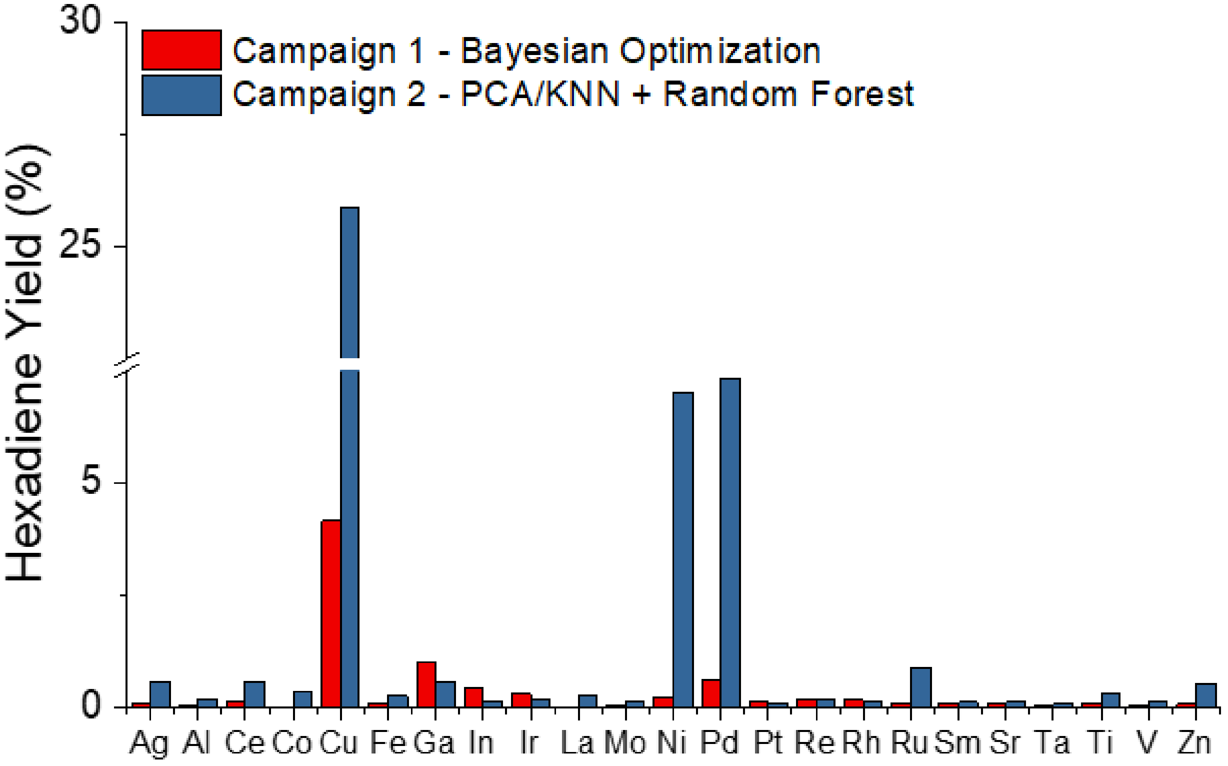

Figure S10. Comparison of maximum hexadiene yield achieved for each metal from campaign 1 to campaign 2.

2. ICP Weight Loadings. Metal analysis was performed at the Northwestern University Quantitative Bio-element Imaging Center. Quantification of Cu, Ni, Pd, Fe, Al, Ti, Ga, Co, Pt, Ag, Re, Ru, Sm, Zn, Ir, Ce, V, La, Ru, Sr, Ta, Mo and In was accomplished using ICP-OES of acid-digested samples and the actual weight loadings for a subset of the data is given in Table S4.

Table S4. ICP weight loadings for a subset of the catalyst compositions with nominal loading of 1 wt%.

Element	wt (%)
Ag	0.38
Al	4.40
Ce	2.45
Co	2.26
Fe	1.48
La	3.89
Ni	3.14
Re	0.13
Sm	3.26
Sr	4.06
V	2.94
Zn	5.49
Ga	0.19
Cu	1.52
In	6.27
Ir	0.06
Pd	2.57
Pt	0.07
Rh	0.04
Ru	1.48
Ta	2.52
Ti	3.24
Mo	5.08

3. Mass Balance

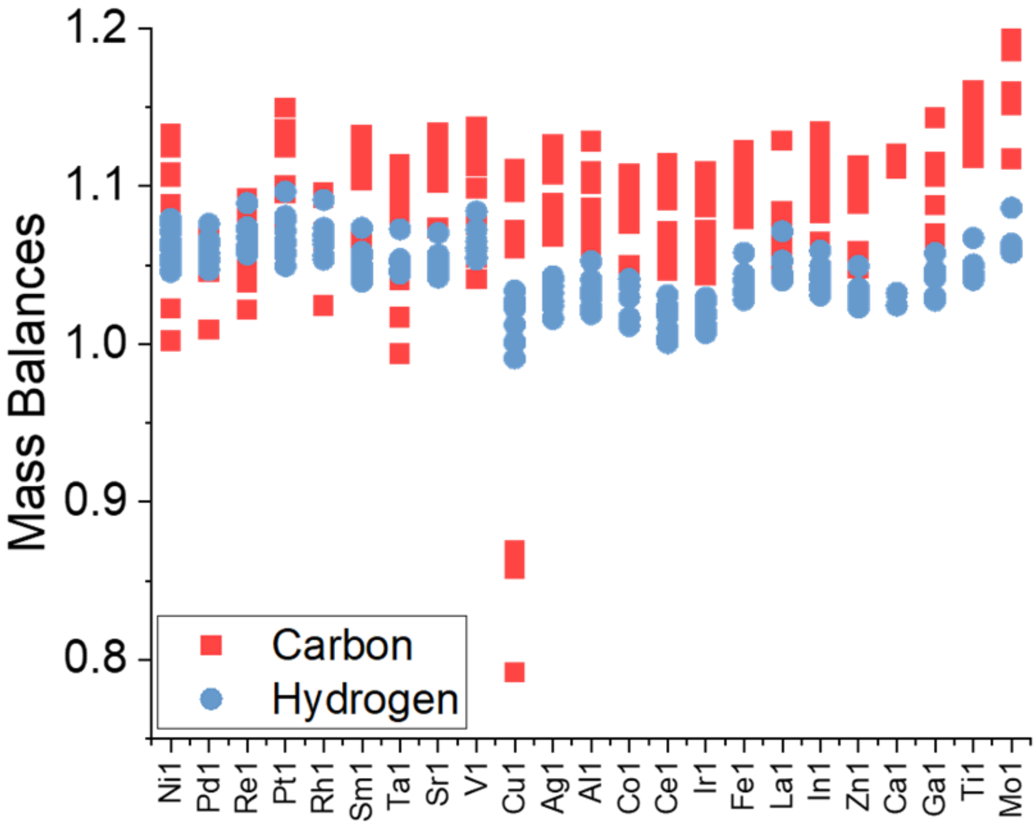

Figure S11. Carbon and Hydrogen balance for each metal with nominal 1 wt% ran in 40% H_2 in balance 2% propyne/Ar, 2,000 mL/min/g_{cat} and 1 bar between 100-200 °C. The average C balance for all data points is 1.06±0.11 and the average H balance for all data points is 1.04 ± 0.02. A hydrogen balance of near unity for catalysts indicates that higher order oligomers did not form that were not accounted for in the GC. Carbon deposits on Cu catalysts were found by Raman spectroscopy as well as along the walls of the reactor, justifying why the carbon balance was less than 100%.

4. Powder X-ray Diffraction of Bare NU-1000 vs. Cu/NU-1000

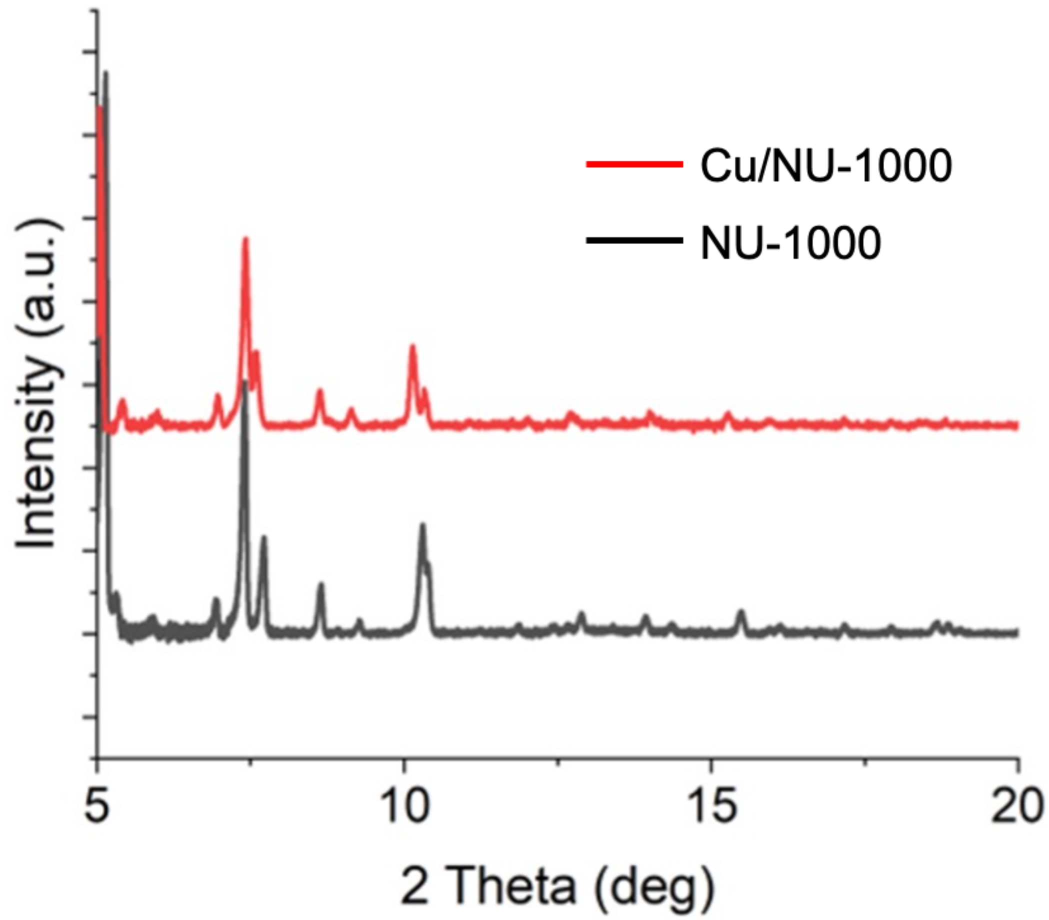

Figure S12. PXRD of bare NU-1000 and Cu/NU-1000.

5. Stability Test

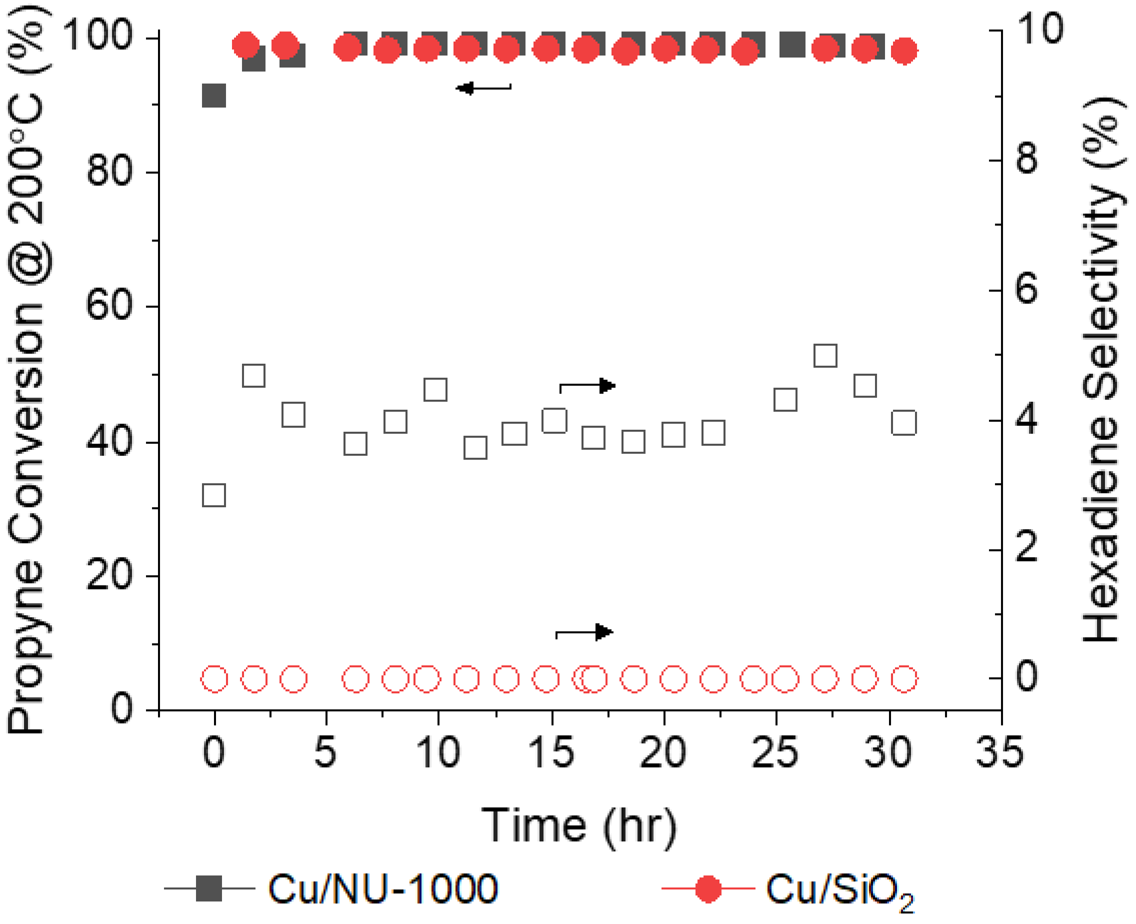

Figure S13. Stability (solid markers) and hexadiene selectivity (hollow markers) of Cu/NU-1000 (black) and Cu/SiO_2 (red) over 33 hours. Reaction conditions: 40% H_2 in balance 2% propyne/Ar, 2,000 mL/min/g_{cat}, T = 200 °C, P = 1 atm. Catalysts contain a nominal loading of 1wt%.

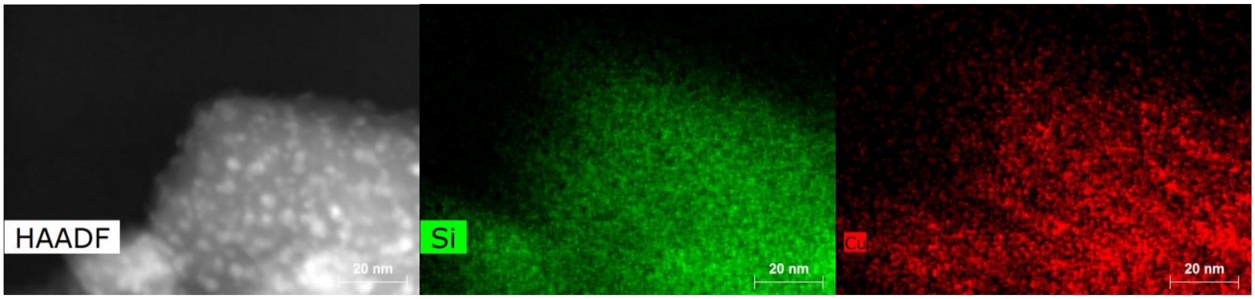

Figure S14. High-angle annular dark-field scanning transmission electron microscopy (HAADF-STEM) of Cu/SiO_2 after reaction.

6. Raman Spectroscopy

Raman Methodology. The data was acquired on a Horiba LabRam HR spectrometer equipped on an inverted optical microscope (Nikon Ti-E) with a 632.8 nm HeNe laser light source and a 40X objective. The Raman spectra were acquired using a backscattering geometry setup through which the scattered light was returned through the same 40X objective, transmitted through a beam splitter, dispersed through a 600 g/mm grating, and detected by an EM-CCD detector. The spectral window is between 1050 and 2050 cm^{-1} with a resolution of 1.05 cm^{-1}. The spectra were acquired by averaging 120 exposures of 1 second duration. To avoid laser-induced changes in the coke deposits, the fluence of the excitation source was reduced by a factor of 100. The spectra were plotted following normalization by the maximum peak height. The spectra are vertically offset.

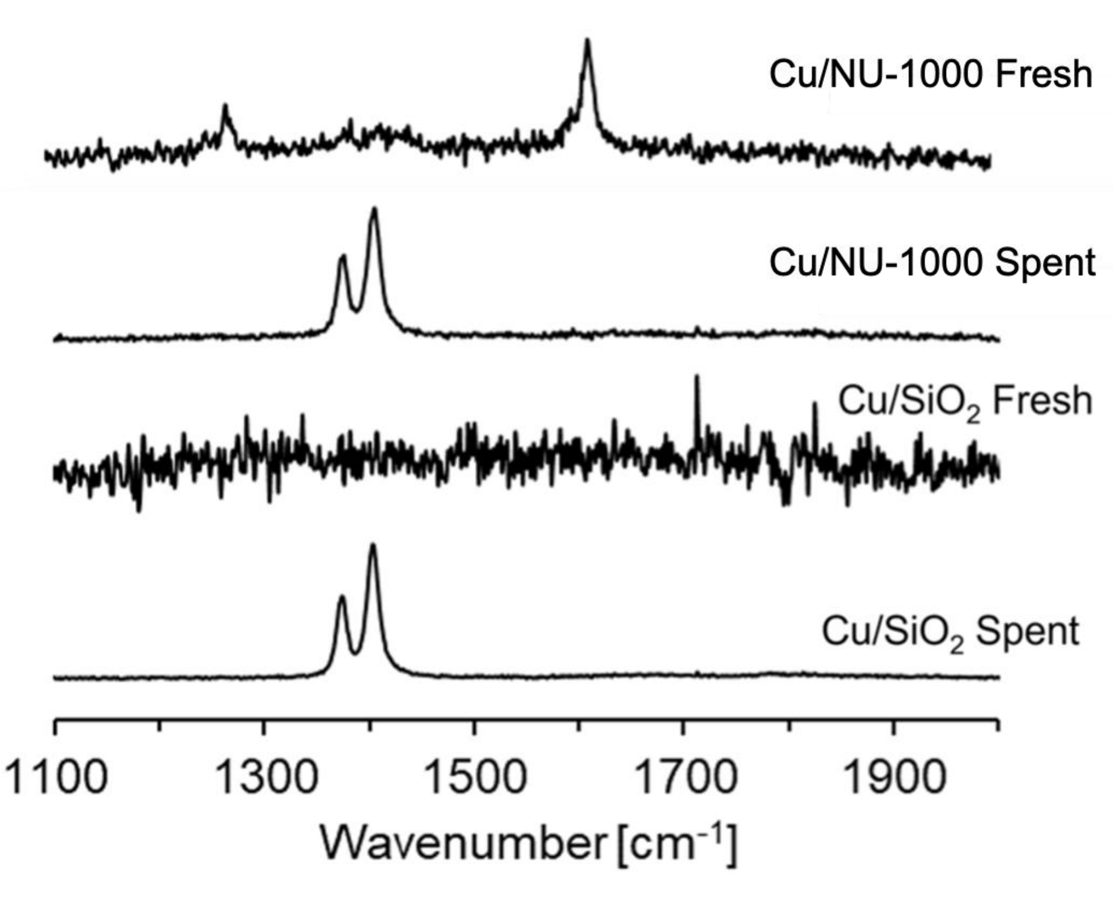

Figure S15. Raman spectra of (from top to bottom) Cu/NU-1000 as synthesized, Cu/NU-1000 after reaction, Cu/SiO_2 as synthesized and Cu/SiO_2 after reaction.

7. DFT Energy Profile

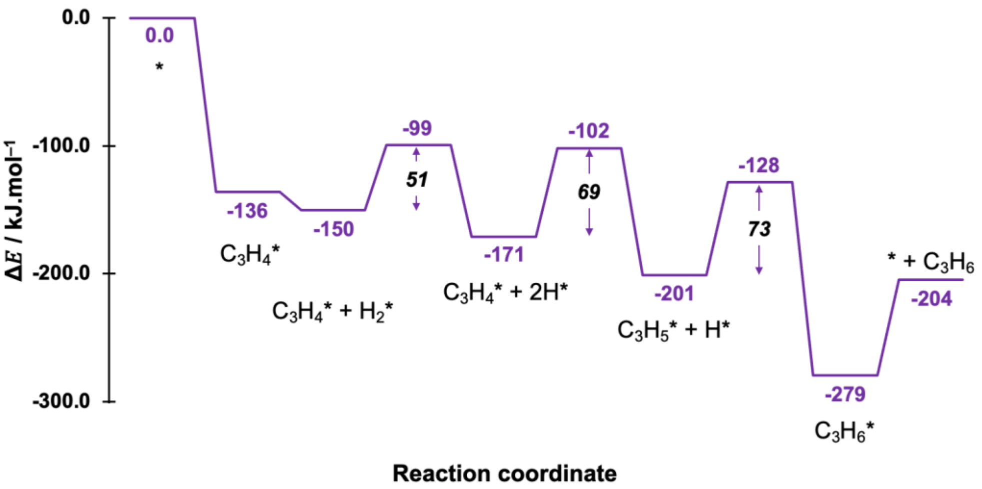

Figure S16. Energy profile (in kJ mol^{-1}) for the hydrogenation of propyne to propene on Cu (111) calculated using PBE-D3 functional. * indicates adsorption on surface sites.

Table S5: Tabulated absolute and relative electronic energies (in eV and kJ mol^{-1} respectively) of the DFT-optimized intermediates and transition states for propyne transformation on Cu(111) using the PBE-D3 density functional.

Structure	$n_{propyne}$	n_{H2}	E (eV)	ΔE (kJ mol^{-1})
H_2 (gas)	0	1	-6.751259	-
Propyne (gas)	1	0	-39.825986	-
Dimerization to hexadiene				
*	0	0	-281.213260	0
C_3H_4*	1	0	-322.450310	-136
C_3H_4* + H_2^*	1	1	-329.344950	-150
TS1	1	1	-328.820690	-99
C_3H_4* + $2H^*$	1	1	-329.564400	-171
TS2	1	1	-328.846870	-102
C_3H_5* + H^*	1	1	-329.873160	-201
C_3H_5* + H^* + C_3H4*	2	1	-371.034230	-330
TS3	2	1	-369.911670	-221
C_6H_9* + H^*	2	1	-371.845490	-408
TS4	2	1	-371.137650	-340
C_6H_{10}*	2	1	-372.797000	-500
product	2	1	-90.434977	-389
Hydrogenation to propene				
TS3b	1	1	-329.11868	-128
$C_3H_6^*$	1	1	-330.68566	-279
product	1	1	-48.696997	-204

Table S6: DFT-optimized geometries of the different transition states (TS) involved in propyne transformation, located using the PBE-D3 density functional. The top view and side view is shown for each TS. The activation barrier for each step (in kJ mol^{-1}) is reported in parentheses.

Reaction	Top view	Side view
TS1<br>$C_3H_4* + H_2* \rightarrow C_3H_4* + 2H*$<br>($\Delta E^{\neq}$ = 51)	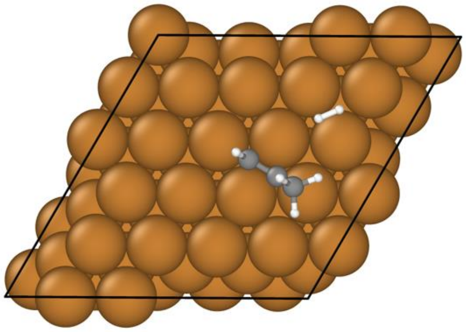	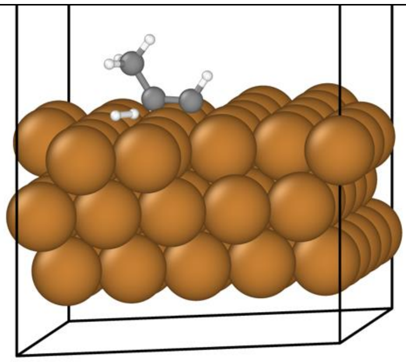
TS2<br>$C_3H_4* + 2H* \rightarrow C_3H_5* + H*$<br>($\Delta E^{\neq}$ = 69)	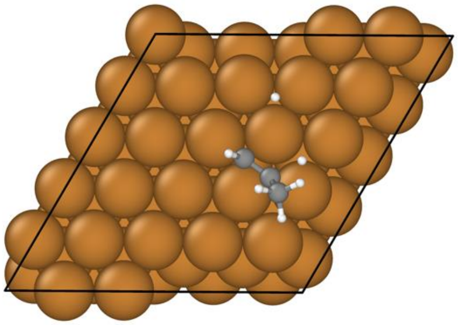	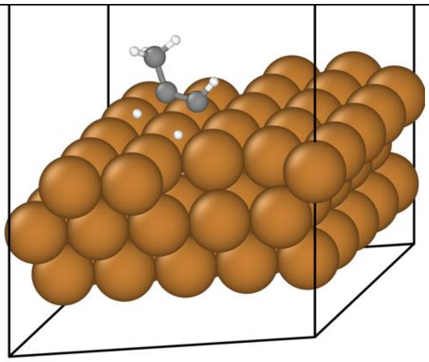
TS3<br>$C_3H_5* + H* + C_3H_4* \rightarrow C_6H_9* + H*$<br>($\Delta E^{\neq}$ = 108)	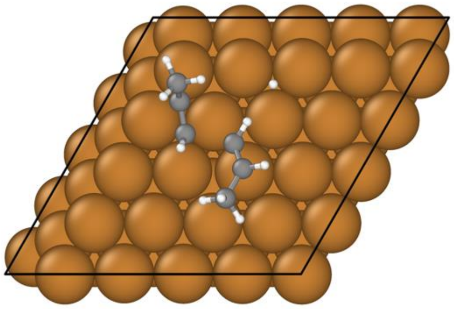	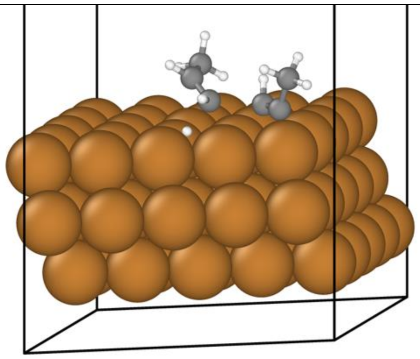

TS4<br><br>$C_6H_9* + H* \rightarrow C_6H_{10}*$<br><br>($\Delta E^{\neq}$ = 68)	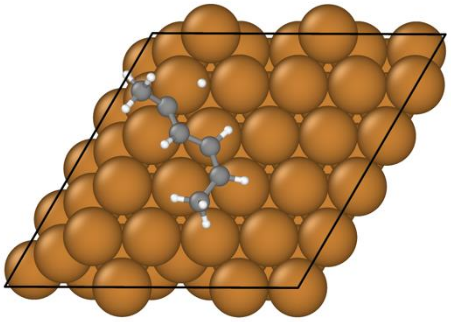	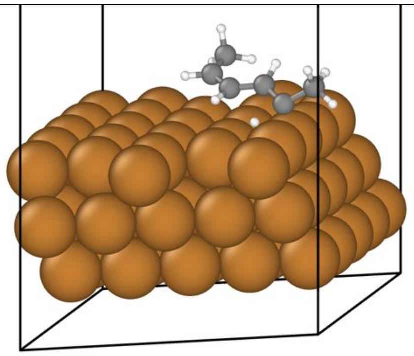
TS3b<br><br>$C_3H_5* + H* \rightarrow C_3H_6*$<br><br>($\Delta E^{\neq}$ = 73)	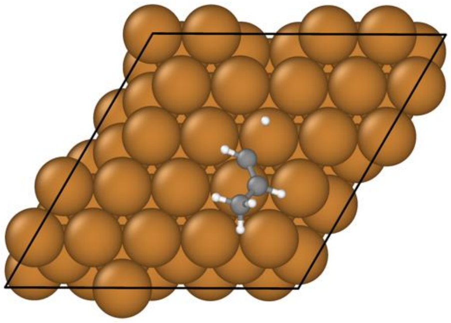	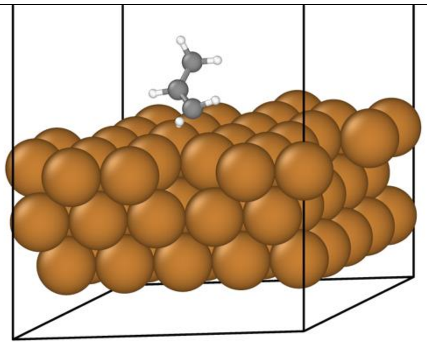

Table S7: Tabulated absolute and relative electronic energies (in Hartree and kJ mol^{-1} respectively) of the DFT-optimized intermediates and transition states for propyne transformation on Cu1-NU-1000 using the M06-L density functional.

**Structure**	n_{propyne}	n_{H2}	***E***	***H***	***G***	**Δ*E***	**Δ*H***	**Δ*G***
			**(Hartree)**			**(kJ mol^{-1})**		
H_2 (gas)	1	0	-1.167207	-1.153870	-1.168691			
propyne (gas)	0	1	-116.550534	-116.490149	-116.519200			
S-1	0	0	-3203.715375	-3203.243921	-3203.383822	0.0	0.0	0.0
S-2	1	0	-3320.299304	-3319.766937	-3319.918283	-87.7	-86.3	-40.1
S-3	1	0.5	-3320.953844	-3320.408720	-3320.559610	-273.9	-256.6	-189.7
S-4	1	1	-3321.534070	-3320.977236	-3321.129336	-265.1	-234.4	-151.3
S-5	2	1	-3438.102362	-3437.483260	-3437.645121	-311.7	-276.1	-142.3
S-56	2	1	-3438.088239	-3437.471229	-3437.632177	-274.6	-244.5	-108.3
S-6	2	1	-3438.159134	-3437.537473	-3437.695954	-460.7	-418.5	-275.8
S-67	2	1	-3438.102595	-3437.485837	-3437.641466	-312.3	-282.9	-132.7
S-7	2	1	-3438.174010	-3437.551541	-3437.710336	-499.8	-455.4	-313.5

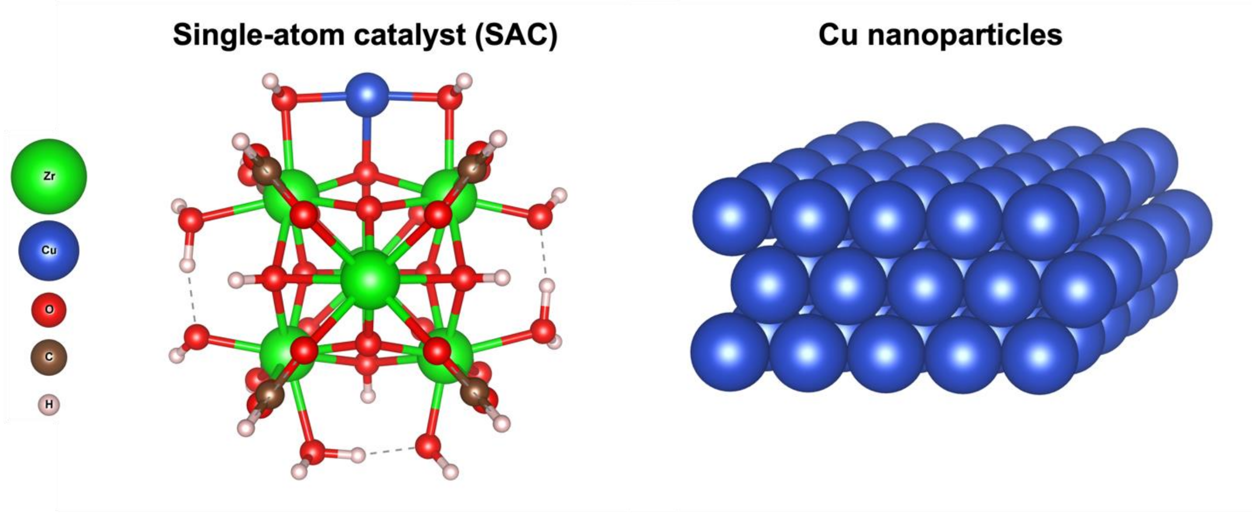

Figure S17. Atomistic models for the single-atom Cu1/NU-1000 (cluster DFT) and the Cu nanoparticles (periodic DFT) used in this work.

8. References

(1) Shahriari, B.; Swersky, K.; Wang, Z.; Adams, R. P.; de Freitas, N. Taking the Human Out of the Loop: A Review of Bayesian Optimization. *Proceedings of the IEEE* **2016**, *104* (1), 148–175.

(2) Shields, B. J.; Stevens, J.; Li, J.; Parasram, M.; Damani, F.; Alvarado, J. I. M.; Janey, J. M.; Adams, R. P.; Doyle, A. G. Bayesian Reaction Optimization as a Tool for Chemical Synthesis. *Nature* **2021**, *590* (7844), 89–96.

(3) Naito, Y.; Kondo, M.; Nakamura, Y.; Shida, N.; Ishikawa, K.; Washio, T.; Takizawa, S.; Atobe, M. Bayesian Optimization with Constraint on Passed Charge for Multiparameter Screening of Electrochemical Reductive Carboxylation in a Flow Microreactor. *Chem. Commun.* **2022**, *58* (24), 3893–3896.

(4) Häse, F.; Roch, L. M.; Kreisbeck, C.; Aspuru-Guzik, A. Phoenics: A Bayesian Optimizer for Chemistry. *ACS Cent. Sci.* **2018**, *4* (9), 1134–1145.

(5) Isbrandt, E. S.; Sullivan, R. J.; Newman, S. G. High Throughput Strategies for the Discovery and Optimization of Catalytic Reactions. *Angew. Chem. Int. Ed.* **2019**, *58* (22), 7180–7191.

(6) Langner, S.; Häse, F.; Perea, J. D.; Stubhan, T.; Hauch, J.; Roch, L. M.; Heumueller, T.; Aspuru-Guzik, A.; Brabec, C. J. Beyond Ternary OPV: High-Throughput Experimentation and Self-Driving Laboratories Optimize Multi-Component Systems. *arXiv:1909.03511 [physics]* **2019**.

(7) Authors, Gp. GPyOpt: A Bayesian Optimization Framework in Python, 2016.

(8) Ward, L.; Agrawal, A.; Choudhary, A.; Wolverton, C. A General-Purpose Machine Learning Framework for Predicting Properties of Inorganic Materials. *npj Comput Mater* **2016**, *2* (1), 1–7.

(9) McCullough, K.; Williams, T.; Mingle, K.; Jamshidi, P.; Lauterbach, J. High-Throughput Experimentation Meets Artificial Intelligence: A New Pathway to Catalyst Discovery. *Phys. Chem. Chem. Phys.* **2020**, *22* (20), 11174–11196.

(10) Bernales, V.; League, A. B.; Li, Z.; Schweitzer, N. M.; Peters, A. W.; Carlson, R. K.; Hupp, J. T.; Cramer, C. J.; Farha, O. K.; Gagliardi, L. Computationally Guided Discovery of a Catalytic Cobalt-Decorated Metal–Organic Framework for Ethylene Dimerization. *J. Phys. Chem. C* **2016**, *120* (41), 23576–23583.
(11) Ye, J.; Gagliardi, L.; Cramer, C. J.; Truhlar, D. G. Computational Screening of MOF-Supported Transition Metal Catalysts for Activity and Selectivity in Ethylene Dimerization. *J. Catal.* **2018**, *360*, 160–167.
(12) Zheng, J.; Ye, J.; Ortuño, M. A.; Fulton, J. L.; Gutiérrez, O. Y.; Camaioni, D. M.; Motkuri, R. K.; Li, Z.; Webber, T. E.; Mehdi, B. L.; Browning, N. D.; Penn, R. L.; Farha, O. K.; Hupp, J. T.; Truhlar, D. G.; Cramer, C. J.; Lercher, J. A. Selective Methane Oxidation to Methanol on Cu-Oxo Dimers Stabilized by Zirconia Nodes of an NU-1000 Metal–Organic Framework. *J. Am. Chem. Soc.* **2019**, *141* (23), 9292–9304.
(13) Shabbir, H.; Pellizzeri, S.; Ferrandon, M.; Soo Kim, I.; A. Vermeulen, N.; K. Farha, O.; Delferro, M.; F. Martinson, A. B.; B. Getman, R. Influence of Spin State and Electron Configuration on the Active Site and Mechanism for Catalytic Hydrogenation on Metal Cation Catalysts Supported on NU-1000: Insights from Experiments and Microkinetic Modeling. *Catal. Sci. Tech.* **2020**, *10* (11), 3594–3602.
(14) Zhao, Y.; Truhlar, D. G. A New Local Density Functional for Main-Group Thermochemistry, Transition Metal Bonding, Thermochemical Kinetics, and Noncovalent Interactions. *J. Chem. Phys.* **2006**, *125* (19), 194101.
(15) Frisch, M. ea; Trucks, G. W.; Schlegel, H. B.; Scuseria, G. E.; Robb, M. A.; Cheeseman, J. R.; Scalmani, G.; Barone, V.; Petersson, G. A.; Nakatsuji, H. Gaussian 16, 2016.
(16) Andrae, D.; Häubermann, U.; Dolg, M.; Stoll, H.; Preu�, H. Energy-Adjustedab Initio Pseudopotentials for the Second and Third Row Transition Elements. *Theoret. Chim. Acta* **1990**, *77* (2), 123–141.
(17) Luchini, G.; Alegre-Requena, J.; Funes-Ardoiz, I.; Paton, R. S. GoodVibes: Automated Thermochemistry for Heterogeneous Computational Chemistry Data. *F1000Research* **2020**, *9*.
(18) Ribeiro, R. F.; Marenich, A. V.; Cramer, C. J.; Truhlar, D. G. Use of Solution-Phase Vibrational Frequencies in Continuum Models for the Free Energy of Solvation. *J. Phys. Chem. B* **2011**, *115* (49), 14556–14562.
(19) Armbrüster, M.; Kovnir, K.; Behrens, M.; Teschner, D.; Grin, Y.; Schlögl, R. Pd−Ga Intermetallic Compounds as Highly Selective Semihydrogenation Catalysts. *J. Am. Chem. Soc.* **2010**, *132* (42), 14745–14747.
(20) Wang, X.; Zhang, X.; Pandharkar, R.; Lyu, J.; Ray, D.; Yang, Y.; Kato, S.; Liu, J.; Wasson, M. C.; Islamoglu, T.; Li, Z.; Hupp, J. T.; Cramer, C. J.; Gagliardi, L.; Farha, O. K. Insights into the Structure–Activity Relationships in Metal–Organic Framework-Supported Nickel Catalysts for Ethylene Hydrogenation. *ACS Catal.* **2020**, *10* (16), 8995–9005.
(21) McCue, A. J.; Anderson, J. A. Recent Advances in Selective Acetylene Hydrogenation Using Palladium Containing Catalysts. *Front. Chem. Sci. Eng.* **2015**, *9* (2), 142–153.
(22) Haug, K. L.; Bürgi, T.; Gostein, M.; Trautman, T. R.; Ceyer, T. Catalytic Hydrogenation of Acetylene on Ni(111) by Surface-Bound H and Bulk H. *J. Phys. Chem. B* **2001**, *105* (46), 11480–11492.
(23) Swamy, K. C. K.; Reddy, A. S.; Sandeep, K.; Kalyani, A. Advances in Chemoselective and/or Stereoselective Semihydrogenation of Alkynes. *Tetrahedron Lett.* **2018**, *59* (5), 419–429.

(24) García-Mota, M.; Gómez-Díaz, J.; Novell-Leruth, G.; Vargas-Fuentes, C.; Bellarosa, L.; Bridier, B.; Pérez-Ramírez, J.; López, N. A Density Functional Theory Study of the 'Mythic' Lindlar Hydrogenation Catalyst. *Theor. Chem. Acc.* **2011**, *128* (4), 663–673.

(25) McCue, A. J.; McRitchie, C. J.; Shepherd, A. M.; Anderson, J. A. Cu/Al_2O_3 Catalysts Modified with Pd for Selective Acetylene Hydrogenation. *J. Catal.* **2014**, *319*, 127–135.

(26) Larsson, M.; Jansson, J.; Asplund, S. The Role of Coke in Acetylene Hydrogenation on Pd/α-Al_2O_3. *J. Catal.* **1998**, *178* (1), 49–57.

(27) Borodziński, A.; Cybulski, A. The Kinetic Model of Hydrogenation of Acetylene–Ethylene Mixtures over Palladium Surface Covered by Carbonaceous Deposits. *Appl. Catal. A: Gen.* **2000**, *198* (1), 51–66.

(28) Vilé, G.; Baudouin, D.; Remediakis, I. N.; Copéret, C.; López, N.; Pérez-Ramírez, J. Silver Nanoparticles for Olefin Production: New Insights into the Mechanistic Description of Propyne Hydrogenation. *ChemCatChem* **2013**, *5* (12), 3750–3759.
